# Principles for characterizing the potential human health effects from exposure to nanomaterials: elements of a screening strategy

**DOI:** 10.1186/1743-8977-2-8

**Published:** 2005-10-06

**Authors:** Günter Oberdörster, Andrew Maynard, Ken Donaldson, Vincent Castranova, Julie Fitzpatrick, Kevin Ausman, Janet Carter, Barbara Karn, Wolfgang Kreyling, David Lai, Stephen Olin, Nancy Monteiro-Riviere, David Warheit, Hong Yang, A report from the ILSI Research Foundation/Risk Science Institute Nanomaterial Toxicity Screening Working Group

**Affiliations:** 1 Department of Environmental Medicine, University of Rochester, 601 Elmwood Avenue, P.O. Box EHSC, Rochester, NY 14642, USA; 2 Project on Emerging Nanotechnologies, Woodrow Wilson International Center for Scholars, 1300 Pennsylvania Avenue, N.W., Washington, DC 20004-3027, USA; 3 MRC/University of Edinburgh Centre for Inflammation Research, ELEGI Colt Laboratory Queen's Medical Research Institute, 47 Little France Crescent, Edinburgh EH16 4TJ, UK; 4 Pathology and Physiology Research Branch, Health Effects Laboratory Division, National Institute for Occupational Safety and Health, 1095 Willowdale Road, Morgantown, WV 26505, USA; 5 Risk Science Institute, ILSI Research Foundation, International Life Sciences Institute, One Thomas Circle, N.W., Suite 900, Washington, DC 20005-5802, USA; 6 Center for Biological and Environmental Nanotechnology, MS-63, P.O. Box 1892, Rice University, Houston, TX 77251-1892, USA; 7 Respiratory/Inhalation Toxicology, Central Product Safety, Procter & Gamble Company, PO Box 538707, Cincinnati, OH 45253-8707, USA; 8 Office of Research and Development, United States Environmental Protection Agency, Ariel Rios Building, Mail Code: 8722F, 1200 Pennsylvania Avenue, N.W., Washington, DC 20460, USA; 9 Project on Emerging Nanotechnologies, Woodrow Wilson International Center for Scholars, 1300 Pennsylvania Avenue, N.W., Washington, DC 20004-3027, USA; 10 Institute for Inhalation Biology & Focus Network: Aerosols and Health, GSF National Research Centre for Environment and Health, Ingolstadter Landstrasse 1, 85764 Neuherberg, Munich, Germany; 11 Risk Assessment Division, Office of Pollution Prevention & Toxics, United States Environmental Protection Agency, 7403M, 1200 Pennsylvania Avenue, N.W., Washington, DC 20460, USA; 12 Center for Chemical Toxicology and Research Pharmacokinetics, College of Veterinary Medicine, North Carolina State University, 4700 Hillsborough Street, Raleigh, NC 27606, USA; 13 DuPont Haskell Laboratory for Health and Environmental Sciences, P.O. Box 50, 1090 Elkton Road, Newark, DE 19714-0050, USA; 14 Department of Chemical Engineering, University of Rochester, Gavett Hall 253, Rochester, NY 14627, USA

## Abstract

The rapid proliferation of many different engineered nanomaterials (defined as materials designed and produced to have structural features with at least one dimension of 100 nanometers or less) presents a dilemma to regulators regarding hazard identification. The International Life Sciences Institute Research Foundation/Risk Science Institute convened an expert working group to develop a screening strategy for the hazard identification of engineered nanomaterials. The working group report presents the *elements *of a screening strategy rather than a detailed testing protocol. Based on an evaluation of the limited data currently available, the report presents a broad data gathering strategy applicable to this early stage in the development of a risk assessment process for nanomaterials. Oral, dermal, inhalation, and injection routes of exposure are included recognizing that, depending on use patterns, exposure to nanomaterials may occur by any of these routes. The three key elements of the toxicity screening strategy are: Physicochemical Characteristics, *In Vitro *Assays (cellular and non-cellular), and *In Vivo *Assays.

There is a strong likelihood that biological activity of nanoparticles will depend on physicochemical parameters not routinely considered in toxicity screening studies. Physicochemical properties that may be important in understanding the toxic effects of test materials include particle size and size distribution, agglomeration state, shape, crystal structure, chemical composition, surface area, surface chemistry, surface charge, and porosity.

*In vitro *techniques allow specific biological and mechanistic pathways to be isolated and tested under controlled conditions, in ways that are not feasible in *in vivo *tests. Tests are suggested for portal-of-entry toxicity for lungs, skin, and the mucosal membranes, and target organ toxicity for endothelium, blood, spleen, liver, nervous system, heart, and kidney. Non-cellular assessment of nanoparticle durability, protein interactions, complement activation, and pro-oxidant activity is also considered.

Tier 1 *in vivo *assays are proposed for pulmonary, oral, skin and injection exposures, and Tier 2 evaluations for pulmonary exposures are also proposed. Tier 1 evaluations include markers of inflammation, oxidant stress, and cell proliferation in portal-of-entry and selected remote organs and tissues. Tier 2 evaluations for pulmonary exposures could include deposition, translocation, and toxicokinetics and biopersistence studies; effects of multiple exposures; potential effects on the reproductive system, placenta, and fetus; alternative animal models; and mechanistic studies.

## 1.0 Introduction

The rapid proliferation of many different engineered nanomaterials presents a dilemma to regulators regarding hazard identification. The screening strategy developed by the International Life Sciences Institute Research Foundation/Risk Science Institute (ILSI RF/RSI) Nanomaterial Toxicity Screening Working Group is an effort to make a significant contribution to the initial hazard identification process for nanomaterial risk assessment.

Engineered nanomaterials are commonly defined as materials designed and produced to have structural features with at least one dimension of 100 nanometers or less. Such materials typically possess nanostructure-dependent properties (e.g., chemical, mechanical, electrical, optical, magnetic, biological), which make them desirable for commercial or medical applications. However, these same properties potentially may lead to nanostructure-dependent biological activity that differs from and is not directly predicted by the bulk properties of the constituent chemicals and compounds. This report outlines the elements of a toxicological screening strategy for nanomaterials as the first step – i.e., hazard identification – in the risk assessment process. Both *in vitro *and *in vivo *methodologies were considered in the development of the screening strategy.

Engineered nanomaterials encompass many forms and are derived from numerous bulk substances. Nanoparticles form a basis for many engineered nanomaterials, and are currently being produced in a wide variety of types for a variety of applications; fullerenes (C_60 _or Bucky Balls), carbon nanotubes (CNT), metal and metal oxide particles, polymer nanoparticles and quantum dots are among the most common.

Engineered nanomaterials are presenting new opportunities to increase the performance of traditional products, and to develop unique new products. "The ability to create unusual nanostructures such as bundles, sheets, and tubes holds promise for new and powerful drug delivery systems, electronic circuits, catalysts, and light-harvesting materials." [1]

Many current efforts are predominantly focused on using relatively simple nanostructured materials such as metal oxide nanoparticles and carbon nanotubes in applications such as high performance materials, energy storage and conversion, self-cleaning surface coatings and stain-resistant textiles. Research into more complex nanomaterials is anticipated to lead to applications such as cellular-level medical diagnostics and treatment and advanced electronics. However, as nanotechnology blurs traditionally rigid boundaries between scientific disciplines, a rapid growth in unanticipated applications is to be expected over the next years and decades.

As new nanotechnology-based materials begin to emerge, it will be essential to have a framework in place within which their potential toxicity can be evaluated, particularly as indicators suggest traditional screening approaches may not be responsive to the nanostructure-related biological activity of these materials.

Several national and international organizations are currently developing standard definitions for comment terms in nanomaterial science including the International Association of Nanotechnology's Nomenclature and Terminology Subcommittee and the American National Standards Institute Nanotechnology Standards Panel (ANSI-NSP). The following key definitions are used throughout this document.

### 1. Nanoparticle

A particle with at least one dimension smaller than 100 nm including engineered nanoparticles, ambient ultrafine particles (UFPs) and biological nanoparticles.

### 2. Engineered/Manufactured Nanoparticle

A particle engineered or manufactured by humans on the nanoscale with specific physicochemical composition and structure to exploit properties and functions associated with its dimensions. Engineered nanoparticles include particles with a homogeneous composition and structure, compositionally and structurally heterogeneous particles (for instance, particles with core-shell structures) and multi-functional nanoparticles (for instance, 'smart' nanoparticles being developed for medical diagnostics and treatment).

### 3. Nanomaterial

A material having a physicochemical structure on a scale greater than typically atomic/ molecular dimensions but less than 100 nm (nanostructure), which exhibits physical, chemical and/or biological characteristics associated with its nanostructure.

### 4. Nanostructured Particle

A particle with a physicochemical structure on a scale greater than atomic/molecular dimensions but less than 100 nm, which exhibits physical, chemical and/or biological characteristics associated with its nanostructure. A nanostructured particle may be much larger than 100 nm. For example, agglomerates of TiO_2 _nanoparticles that are significantly larger than 100 nm in diameter may have a biological activity determined by their nanoscale sub-structure. Other examples include zeolites, meso-porous materials and multifunctional particulate probes.

### 5. Agglomerate/Aggregate

The terms "agglomerate" and "aggregate" are used differently and even interchangeably in different fields. In the context of this report, the term "agglomerate" is used exclusively to describe a collection of particles that are held together by both weak and strong forces, including van der Waals and electrostatic forces, and sintered bonds. In this document, the term is used interchangeably with 'aggregate'. However, the importance of understanding how the binding forces of an agglomerate affect the dispersibility of the component particles under different conditions – in essence how easily the agglomerate de-agglomerates – is noted.

### 6. Nanoporous Material

A material with particles that are larger than 100 nm may have significant structuring on the nanometer size scale, thereby providing properties based upon this smaller structuring that may be toxicologically relevant (e.g., dramatically increased surface area as compared to the bulk). Nanoporous materials, such as zeolites, are a significant class of materials which have porosity in the sub-100 nm size range but whose primary particles may be large.

## 2.0 Objectives and Scope

The objective of the ILSI RSI Nanomaterial Toxicity Screening Working Group, which was convened in February 2005, was to identify the key elements of a toxicity screening strategy for engineered nanomaterials. The group considered potential effects of exposure to nanomaterials by inhalation, dermal, oral, and injection routes; discussed how mechanisms of nanoparticle toxicity may differ from those exhibited by larger particles of the same chemical; and identified significant data needs for designing a robust screening strategy.

The elements of a screening strategy for nanomaterials presented by the Nanomaterial Toxicity Screening Working Group include an evaluation of the physicochemical characteristics and dose metrics; acellular assays; *in vitro *assays for lung, skin, and mucosal membranes; and *in vivo *assays for lung, skin, oral, and injection exposures.

This project was funded by the U.S. Environmental Protection Agency Office of Pollution Prevention and Toxics through a cooperative agreement with the ILSI Research Foundation/Risk Science Institute. It was an outgrowth of another project under the same cooperative agreement that proposed strategies for short-term toxicity testing of fibers [2]. Among the principal conclusions of the latter project were the importance of the physicochemical characterization of the fibers, the value of subchronic (1–3 month) rat inhalation exposure studies, and the typically key role in fiber toxicity of biopersistence of inhaled fibers in the lung and of chronic inflammation leading to cell proliferation and interstitial fibrosis.

## 3.0 Literature Survey

The potential for human and ecological toxicity associated with nanomaterials and ultrafine particles is a growing area of investigation as more nanomaterials and products are developed and brought into commercial use. To date, few nanotoxicology studies have addressed the effects of nanomaterials in a variety of organisms and environments. However, the existing research raises some concerns about the safety of nanomaterials and has led to increased interest in studying the toxicity of nanomaterials for use in risk assessment and protection of human health and the environment. A new field of nanotoxicology has been developed to investigate the possibility of harmful effects due to exposure to nanomaterials [3]. Nanotoxicology also encompasses the proper characterization of nanomaterials used in toxicity studies. Characterization has been important in differentiating between naturally occurring forms of nanomaterials, nano-scale byproducts of natural or chemical processes, and manufactured (engineered) nanomaterials. Because of the wide differences in properties among nanomaterials, each of these types of nanoparticles can elicit its own unique biological or ecological responses. As a result, different types of nanomaterials must be categorized, characterized, and studied separately, although certain concepts of nanotoxicology based on the small size, likely apply to all nanomaterials.

As materials reach the nanoscale, they often no longer display the same reactivity as the bulk compound. For example, even a traditionally inert bulk compound, such as gold, may elicit a biological response when it is introduced as a nanomaterial [4]. New approaches for testing and new ways of thinking about current materials are necessary to provide safe workplaces, products, and environments as the manufacturing of nanomaterials and products increases and, as a result, exposure to nanomaterials increases. The diverse routes of exposure, including inhalation, dermal uptake, ingestion, and injection, can present unique toxicological outcomes that vary with the physicochemical properties of the nanoparticles in question.

The earliest studies investigating the toxicity of nanoparticles focused on atmospheric exposure of humans and environmentally relevant species to heterogeneous mixtures of environmentally produced ultrafine particulate matter (having a diameter <100 nm). These studies examined pulmonary toxicity associated with particulate matter deposition in the respiratory tract of target organisms [5-15]. Epidemiological assessments of the effects of urban air pollution exposure focusing on particulate matter produced as a byproduct of combustion events, such as automobile exhaust and other sources of urban air pollution, showed a link in test populations between morbidity and mortality and the amount of particulate matter [16-19]. Some researchers have found an increased risk of childhood and adult asthma correlated to environmental exposure to ultrafine particulate matter in urban air [20-22]. However, other research does not indicate the same correlation [23-25].

Laboratory-based studies have investigated the effects of a large range of ultrafine materials through *in vivo *exposures using various animal models as well as cell-culture-based *in vitro *experiments. To date, animal studies routinely show an increase in pulmonary inflammation, oxidative stress, and distal organ involvement upon respiratory exposure to inhaled or implanted ultrafine particulate matter [7,11,26-30]. Tissue and cell culture analysis have also supported the physiological response seen in whole animal models and yielded data pointing to an increased incidence of oxidative stress, inflammatory cytokine production, and apoptosis in response to exposure to ultrafine particles [31-37]. These studies have also yielded information on gene expression and cell signaling pathways that are activated in response to exposure to a variety of ultrafine particle species ranging from carbon-based combustion products to transition metals. Polytetrafluoroethylene fumes in indoor air pollution are nano-sized particles, highly toxic to rats [38]. They elicit a severe inflammatory response at low inhaled particle mass concentrations, suggestive of an oxidative injury [39-41].

In contrast to the heterogeneous ultrafine materials produced incidentally by combustion or friction, manufactured nanomaterials can be synthesized in highly homogenous forms of desired sizes and shapes (e.g., spheres, fibers, tubes, rings, planes). Limited research on manufactured nanomaterials has investigated the interrelationship between the size, shape, and dose of a material and its biological effects, and whether a unique toxicological profile may be observed for these different properties within biological models.

Typically, the biological activity of particles increases as the particle size decreases. Smaller particles occupy less volume, resulting in a larger number of particles with a greater surface area per unit mass and increased potential for biological interaction [42-46]. Recent studies have begun to categorize the biological response elicited by various nanomaterials both in the ecosystem and in mammalian systems. Although most current research has focused on the effect of nanomaterials in mammalian systems, some recent studies have shown the potential of nanomaterials to elicit a phytotoxic response in the ecosystem. In the case of alumina nanoparticles, one of the US market leaders for nano-sized materials, 99.6% pure nanoparticles with an average particle size of 13 nm were shown to cause root growth inhibition in five plant species [46].

Toxicological studies of fibrous and tubular nanostructures have shown that at extremely high doses these materials are associated with fibrotic lung responses and result in inflammation and an increased risk of carcinogenesis. Single-walled carbon nanotubes (SWCNT) have been shown to inhibit the proliferation of kidney cells in cell culture by inducing cell apoptosis and decreasing cellular adhesive ability. In addition, they cause inflammation in the lung upon instillation [26,33,47-49]. Multi-walled carbon nanotubes (MWCNT) are persistent in the deep lung after inhalation and, once there, are able to induce both inflammatory and fibrotic reactions [47].

Dermal exposure to MWCNT has been modeled through cell culture and points to the nanoparticles' ability to localize within and initiate an irritation response in target epithelial cells [50]. Proteomic analysis conducted in human epidermal keratinocytes exposed to MWCNT showed both increased and decreased expression of many proteins relative to controls. These protein alterations suggested dysregulation of intermediate filament expression, cell cycle inhibition, altered vesicular trafficking/exocytosis and membrane scaffold protein down-regulation [50,51]. In addition, gene expression profiling was conducted on human epidermal keratinocytes exposed to SWCNT that showed a similar profile to alpha-quartz or silica. Also, genes not previously associated with these particulates before from structural protein and cytokine families were significantly expressed [52]. Dosing keratinocytes and bronchial epithelial cells *in vitro *with SWCNT has been shown to result in increases in markers of oxidative stress [50,53,54].

Charge properties and the ability of carbon nanoparticles to affect the integrity of the blood-brain barrier as well as exhibit chemical effects within the brain have also been studied. Nanoparticles can overcome this physical and electrostatic barrier to the brain. In addition, high concentrations of anionic nanoparticles and cationic nanoparticles are capable of disrupting the integrity of the blood-brain barrier. The brain uptake rates of anionic nanoparticles at lower concentrations were greater than those of neutral or cationic formulations at the same concentrations. This work suggests that neutral nanoparticles and low concentration anionic nanoparticles can serve as carrier molecules providing chemicals direct access to the brain and that cationic nanoparticles have an immediate toxic effect at the blood-brain barrier [55,56].

Tests with uncoated, water soluble, colloidal C_60 _fullerenes have shown that redox-active, lipophilic carbon nanoparticles are capable of producing oxidative damage in the brains of aquatic species [55]. The bactericidal potential of C_60 _fullerenes was also observed in these experiments. This property of fullerenes has possible ecological ramifications and is being explored as a potential source of new antimicrobial agents [57-59].

Oxidative stress as a common mechanism for cell damage induced by nano- and ultrafine particles is well documented; fullerenes are model compounds for producing superoxide. A wide range of nanomaterial species have been shown to create reactive oxygen species both *in vivo *and *in vitro*. Species which have been shown to induce free radical damage include the C_60 _fullerenes, quantum dots, and carbon nanotubes [30,60-66]. Nanoparticles of various sizes and chemical compositions are able to preferentially localize in mitochondria where they induce major structural damage and can contribute to oxidative stress [65].

Quantum dots (QDs) such as CdSe QDs have been introduced as new fluorophores for use in bioimaging. When conjugated with antibodies, they are used for immunostaining due to their bright, photostable fluorescence.

To date, there is not sufficient analysis of the toxicity of quantum dots in the literature, but some current studies point to issues of concern when these nanomaterials are introduced into biological systems. Recently published research indicates that there is a range of concentrations where quantum dots used in bioimaging have the potential to decrease cell viability, or even cause cell death, thus suggesting that further toxicological evaluation is urgently needed [67,68]. While it is well known that bulk cadmium selenide (CdSe) is cytotoxic, it has been suggested that CdSe quantum dots are cytocompatible, and safe for use in whole animal studies. This postulate is based in part on the use of protecting groups around the CdSe core of the quantum dot. These coatings have been shown to be protective, but their long-term stability has not been evaluated thoroughly. Recent studies exploring the cytotoxicity of CdSe-core quantum dots in primary hepatocytes as a liver model found that these quantum dots were acutely toxic under certain conditions. The cytotoxicity correlates with the liberation of free Cd^2+ ^ions due to deterioration of the CdSe lattice. These data suggest that quantum dots can be rendered nontoxic initially for *in vivo *use when appropriately coated. However, the research also highlights the need to further explore the long-term stability of the coatings used, both *in vivo *and exposed to environmental conditions [69].

The range of approaches and methods used to reach conclusions regarding the effects of manufactured nanomaterials and ultrafine particles has led to different results. This inconsistency indicates a need for standardized tests in order to get comparable results in screening nanomaterials for potential adverse effects. As the field of nanotoxicology continues to grow, standard toxicology tests will aid those entering the field and allow for better comparisons and conclusions in determining the toxic effects of nanomaterials.

## 4.0 Elements of a screening strategy for nanomaterials

**Figure 1:**
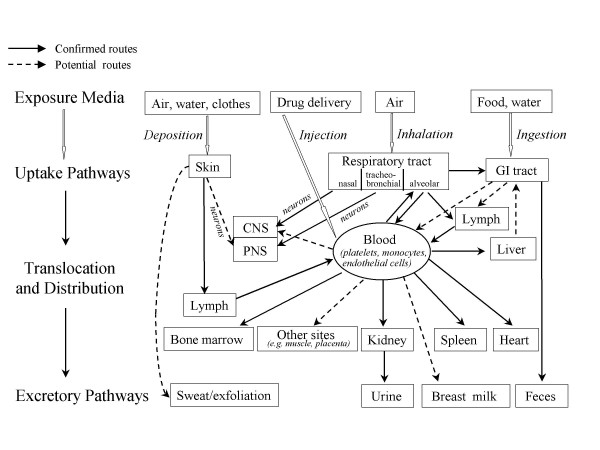
Biokinetics of Nano-sized Particles. While many uptake and translocation routes have been demonstrated, others still are hypothetical and need to be investigated. Largely unknown are translocation rates as well as accumulation and retention in critical target sites and their underlying mechanisms. These as well as potential adverse effects will be largely dependent on physicochemical characteristics of the surface and core of nano-sized particles. Both qualitative and quantitative changes in nano-sized particles' biokinetics in a diseased or compromised organism need also to be considered. Reproduced with permission from Environmental Health Perspectives.

While the nanostructure-dependent properties of many engineered nanomaterials may place them in the category of potential hazards, the direct risk they present to human health will depend on the probability of exposures occurring, and the extent to which materials entering the body exhibit behavior associated with their nanostructure. Figure 1[70]; Biokinetics of Nano-sized Particles; While many uptake and translocation routes have been demonstrated, others still are hypothetical and need to be investigated. Largely unknown are translocation rates as well as accumulation and retention in critical target sites and their underlying mechanisms. These as well as potential adverse effects will be largely dependent on physicochemical characteristics of the surface and core of nano-sized particles. Both qualitative and quantitative changes in nano-sized particles biokinetics in a disease or compromised organism need also to be considered.

In many cases, nanostructured materials will be components of large-scale products such as nano-composites, surface coatings and electronic circuits, and the potential for direct exposure will be negligible. However, if nanostructured materials may enter the body, toxicity screening strategies are required to ascertain the potential risk they present.

Nanoparticles are an obvious form of engineered nanomaterial presenting a significant exposure potential, because they can be readily deposited in the lungs or on the skin, and potentially translocate within the body. However, agglomerates of nanoparticles from a few hundred nanometers to a few micrometers in diameter may also be inhaled, ingested or deposited on the skin, and may have the potential to express toxicity associated with their nanostructure. Similarly, it is conceivable that nanostructured particles of a few micrometers in diameter and below (such as fragments of a nano-composite or a nanostructured surface coating) may exhibit nanostructure-dependent biological properties. In each of these cases, exposure potential exists for materials in air and in liquid suspensions or slurries.

In this section, three key aspects of toxicity screening strategies are addressed: characterization of nanomaterials, *in vitro *screening strategies and *in vivo *screening strategies (covering inhalation, dermal, ingestion, and injection exposure routes). Screening strategies are developed around nanoparticles, but are relevant to all engineered nanomaterials that are capable of entering the body through inhalation, ingestion, dermal penetration, or injection and expressing biological activity which is associated with their nanostructure.

### 4.1 Physicochemical Characterization

#### 4.1.1 Introduction

Unlike gases, liquids and many solid materials, the desirable properties of engineered nanomaterials closely depend on size, shape and structure (both physically and chemically) at the nanoscale. Similarly, there is a strong likelihood that biological activity will depend on physicochemical parameters not usually considered in toxicity screening studies. Although quantitative toxicity studies on engineered nanomaterials are still relatively sparse, published data on fullerenes, single walled carbon nanotubes, nanoscale metal oxides such as TiO_2 _and nanometer-diameter low solubility particles, support the need to carefully consider how nanomaterials are characterized when evaluating potential biological activity [62,71-75]. Respirable fibers present perhaps the closest analogy to a material that is not fully characterized by mass and chemical composition alone. However, the diversity and complexity of nanomaterials suggests that the level of characterization appropriate to toxicity screening tests will be commensurately more sophisticated.

Until the mechanistic associations between nanomaterial characteristics and toxicity are more fully understood, it will be necessary to ensure that all nanomaterial characteristics that are potentially significant are measured or can be derived in toxicity screening tests. In particular, in as far as it is possible; it is desirable to collect sufficient information to allow retrospective interpretation of toxicity data in the light of new findings. In this context, identifying a set of characterization criteria for nanomaterial toxicity screening presents a significant challenge. Clearly, the ideal of characterizing every possible aspect of a test material, while laudable, is impractical. In this document, we have therefore focused on the context under which characterization takes place and the minimum set of characterization parameters we consider essential within that context. Essential parameters have been supplemented with those considered desirable and those considered of interest but optional within a screening study. The two overarching characterization contexts discussed are human exposure studies and *in vitro*/*in vivo *studies. In the case of the latter, we consider material characterization after administration, characterization at the point of administration and characterization of the bulk material as produced or supplied. The relative importance of characterizing dose against different physical metrics during inhalation exposures is also discussed. Recommendations are subsequently made on physicochemical characterizations for nanomaterial toxicity screening tests and characterization methods capable of providing the recommended information.

#### 4.1.2 Framework for Material Characterization

Material characterization for toxicity screening studies is most appropriately considered in the context of the studies being undertaken. Requirements for *in vitro *and *in vivo *screening studies will differ according to the material delivery route or method. Additionally, understanding human exposures in the context of developing appropriate screening studies will present a further set of characterization requirements. Four screening study contexts are proposed, and characterization recommendations are developed within these contexts:

• Human exposure characterization

• Characterization of material following administration

• Characterization of administered material

• Characterization of as-produced or supplied material

##### Human Exposure Characterization

Where exposure to a specific material is known to occur or is anticipated, exposure studies are desirable in developing and selecting appropriate toxicity screening tests. At present engineered nanomaterials are predominantly at the research or pre-production stage, and there are relatively few environments where exposures are known to occur. However, if commercialization of products using nanomaterials develops as anticipated, the potential for exposure is likely to increase dramatically over the coming decade. Therefore, estimates of future use and potential human exposures should be considered in the development of toxicity screening.

##### Nanomaterial Characterization after Administration

Characterizing delivered nanomaterial after administration in a test system or model provides the highest quality of data on dose and material properties that are related to observed responses, but this is limited by current methodological capabilities. Characterization after administration is particularly advantageous where the possibility of physicochemical changes in the material before and after administration exists. Examples of potential changes include aggregation state, physisorption or chemisorption of biomolecules and biochemically-induced changes in surface chemistry. In addition, possible physicochemical changes as a result of nanomaterial interactions with the surrounding biological systems such as rapid dissolution of water- or lipid soluble fractions of the nanomaterial need to be carefully considered. While characterization after administration is considered an ideal to work towards, it is recognized that in many cases, characterization at the point of administration will be a more realistic and feasible option. It is also recognized that in many cases, characterization at the point of administration will be essential for the intercomparison of studies, irrespective of whether characterization after administration is carried out.

##### Characterization of Administered Material

Characterization of administered material in toxicity screening studies is fundamental. This approach addresses potential physicochemical changes between the bulk material and the administered material (such as agglomeration state) and allows more robust causal associations between the material and observed responses to be developed. However, given the strong sensitivity of many nanomaterial properties to their local environment, it should be noted that biologically relevant changes in the physicochemical nature of a nanomaterial between administration and deposition may have a significant impact on observed responses in some instances.

##### Characterization of As-Produced or Supplied Material

Characterization of nanomaterials as-produced or as-supplied represents the most direct approach to obtaining physicochemical information and may provide useful baseline data on the material under test. Most engineered nanomaterials have a functionality based on their physicochemistry. It is therefore likely that information of relevance to toxicity screening studies will be available from suppliers or producers in many cases. However, due to the current lack of accepted nanomaterial characterization standards, it is strongly recommended that wherever possible, independent characterization of test nanomaterials be conducted.

Characterization of supplied nanomaterial may not appropriately represent physicochemical properties of the material during or following administration. For this reason exclusive reliance on this approach is discouraged, and is only recommended where characterization of material during or after administration is clearly not feasible.

#### 4.1.3 Key Characteristics

Previous studies of asbestos and other fibers have shown that the dimension, durability and dose (the three D's) of fibrous particles are key parameters with respect to their pathogenicity. In general, fibers with a smaller diameter will penetrate deeper in the lungs. Long fibers (longer than the diameter of alveolar macrophages) stimulate macrophages to release inflammatory mediators and will only be cleared slowly. In addition to fiber length, chemical factors play an important role in fiber durability and biopersistence; fibers with high alkali or alkali earth oxide contents and low contents of Al_2_O_3_, Fe_2_O_3_, TiO_2 _tend to have low durability and hence low biopersistence [76]. On the other hand, studies of mineral particles have demonstrated that the toxic and carcinogenic effects are, in some cases, related to the surface area of inhaled particles and their surface activity [77,78]. Particle surface characteristics are considered to be key factors in the generation of free radicals and reactive oxygen species formation and in the development of fibrosis and cancer by quartz (crystallized silica) [77].

The unusual properties of nanomaterials are predominantly associated with their nanometer-scale structure, size and structure-dependent electronic configurations and an extremely large surface-to-volume ratio relative to bulk materials. Particles in the nanosize range can deposit in all regions of the respiratory tract including the distal lungs. Due to their small size, nanoparticles may pass into cells directly through the cell membrane or penetrate between or through cells and translocate to other parts of the body. Limited data have suggested possible translocation of inhaled nanoparticles to the nervous system and other organs/tissues [79-81].

The size of nanoparticles alone may not be the critical factor determining their toxicity; the overall number and thus the total surface area may also be important. As a particle decreases in size, the surface area increases (per unit mass only; if you normalize to number of particles, the surface area decreases) and a greater proportion of atoms/molecules are found at the surface compared to those inside. Thus, nanoparticles have a much larger surface area per unit mass compared with larger particles. The increase in the surface-to-volume ratio results in the increase of the particle surface energy which may render them more biologically reactive.

Nano-scale materials are known to have various shapes and structures such as spheres, needles, tubes, plates, etc. Nanoporous materials are materials with defined pore-sizes in the nanometer range. The effects of the shape on the toxicity of nanomaterials are unknown. The shape of nanomaterials may have effects on the kinetics of deposition and absorption in the body. The results of a recent *in vitro *cytotoxicity study appear to suggest that single-wall nanotubes are more toxic than multi-wall nanotubes [82].

Chemical composition is another important parameter for the characterization of nanomaterials, which comprise nearly all substance classes, e.g., metal/metal oxides, compounds, polymers as well as biomolecules. Some nanomaterials can also be a combination of the above components in core-shell or other complex structures. Dependent on the particle surface chemistry, reactive groups on a particle surface will certainly modify the biological effects. Under ambient conditions, some nanoparticles can form aggregates or agglomerates. These agglomerates have various forms, from dendritic structure to chain or spherical structures. To maintain the characteristics of nanoparticles, they are often stabilized with coatings or derivative surface to prevent agglomeration. The properties of nanoparticles can be significantly altered by surface modification and the distribution of nanoparticles in the body strongly depends upon the surface characteristics. Changes of surface properties by coating of nanoparticles to prevent aggregation or agglomeration with different types and concentrations of surfactants have been shown to change their body distribution and the effects on the biological systems significantly [83,84].

Therefore, it is recommended that the following physicochemical properties of the test materials should be characterized:

• Size distribution

• Agglomeration state

• Shape

• Crystal structure

• Chemical composition – including spatially averaged (bulk) and spatially resolved heterogeneous composition

• Surface area

• Surface chemistry

• Surface charge

• Porosity

#### 4.1.4 Dose Metrics

In any toxicity screening study, careful consideration should be given to the metric used to quantify dose. Although response may be associated with a wide range of physicochemical characteristics, measuring dose against a physical metric of **mass**, **surface area **or **particle number **for a well-characterized material will enable quantitative interpretation of data. Appropriate selection of the dose metric will depend on the hypothesized parameter most closely associated with anticipated response or the metric which may be most accurately measured. It is strongly recommended that in all cases, sufficient information is collected to enable dose against *all three primary physical metrics *to be derived. This may be achieved where the relationships between nanomaterial mass, surface area and particle number concentration are known, or where measurements of particle size distribution are made that enable derivation of all three dose metrics. Where nanomaterials are administered in a liquid medium, such as in the technique of intratracheal instillation or pharyngeal aspiration, the nature and amount of material within the suspension should be fully characterized before delivery in terms of number, surface area and mass concentration. Inhalation studies present additional challenges of measuring dose over time, and require both on-line (time resolved) and off-line analysis.

Off-line **mass **concentration measurements using filter-based methods offer continuity with standard inhalation studies and are recommended as an essential component of inhalation nanomaterial screening tests. Likewise, on-line mass concentration measurements are recommended as an essential component of inhalation studies. Gravimetric and/or chemical analysis of filter samples will provide the most accurate characterization of exposure in many cases when compared to off-line surface area and number concentration analyses. With appropriate additional information, such measurements may be used to calculate aerosol surface area or number concentration. However, the diameter cubed relationship between particle size and mass can lead to large errors when transforming from mass to number concentration if the size distribution is broad or there are small numbers of excessively large particles present. On-line mass-concentration measurements using instruments such as the Tapered Element Oscillating Microbalance (TEOM^®^) potentially offer high precision and good accuracy [85], although they are susceptible to errors where the sampled aerosol contains volatile components. On-line photometric mass concentration methods are generally good for monitoring the temporal stability of aerosol and providing a real-time indication of mass exposure, although they are relatively insensitive to particles smaller than approximately 0.5 μm in diameter [86]. However, in general more appropriate methods should be used for providing real-time measurements of number and surface-area exposure [85,87-89].

Aerosol **size distribution **measurements enable reasonably good calculation of exposure against all three physical metrics, if parameters such as particle shape and density are known. Off-line size distribution measurement methods such as Transmission Electron Microscopy (TEM) analysis offer detailed information on this distribution but are extremely time consuming, and frequently limited by the collection techniques and, in the case of TEM analysis, inference of 3-dimensional structure from 2-dimensional images. On-line size measurement techniques such as Differential Mobility Analysis [90] are capable of measuring aerosol size distribution with a time resolution of tens of seconds. Aerosol number concentration between given particle diameters is easily derived from aerosol size distribution measurements, although interpretation of such data in terms of mass or surface-area dose requires additional information on particle characteristics such as shape and density. It is recommended that for each nanoparticle type, size distribution measurement techniques be validated against TEM analysis.

Off-line **surface area **characterization is possible using isothermal gas-adsorption, although techniques suited to filter samples need to be employed. There is also some possibility that the surface area of the collected material will differ from that of the airborne material due to compaction and surface occlusion. However, the extent to which this may occur is not well understood. Published studies have shown a good correlation between off-line surface area measurement and biological response [91], suggesting that errors associated with collection and subsequent analysis can be small. This holds particularly for insoluble particles; ideally surface area measurements would be required on the insoluble core of a nanomaterial after its water-and/or lipid soluble compounds have been dissolved from the particle surface. Aerosol diffusion charging has been shown to provide a measure of surface area on-line where the charging rate is low [89], and a small number of aerosol diffusion chargers are commercially available. These devices have been shown to measure aerosol surface area well for particles smaller than 100 nm in diameter [87]. At larger diameters, measured surface area progressively underestimates aerosol surface area. In particular, the surface area of porous particle structures as well as that of highly aggregated particles will generally not be determined. Data have been published on a particular aerosol diffusion charger indicating that it provides a measure of aerosol surface area dose in the lungs, as opposed to aerosol surface area exposure [92]. While on-line aerosol surface area measurements are desirable during inhalation exposure studies, uncertainties associated with current techniques suggest caution when interpreting such measurements.

**Number concentration **may be measured on-line with relative ease using instruments such as Condensation Particle Counters [88]. Although it is not clear how biologically relevant number concentration is as a dose metric, the ease with which such measurements are made and their value in tracking temporal changes in exposure lead to their being recommended as essential in inhalation studies.

Table 1 summarizes recommendations for measuring exposure during inhalation studies.

**Table 1:** Recommendations for measuring exposure during inhalation studies

**Metric**	**Measurement Recommendation**
Mass – off-line	E (coupled with on-line)
Mass – on-line	E
Size distribution – off line	E
Size distribution – on line	E/D
Surface area – off line	O
Surface area – on line	O
Number – off line	N
Number – on line	E

E: These measurements are considered to be essential.

D: These measurements are considered to provide valuable information, but are not recommended as essential due to constraints associated with complexity, cost and availability.

O: These measurements are considered to provide valuable but non-essential exposure information.

N: These measurements are not considered to be of significant value to inhalation studies.

#### 4.1.5 Characterization Prioritization

In developing recommendations on material characterizations for nanomaterial toxicity screening studies, three specific factors have been taken into consideration: the context within which a material is being evaluated, the importance of measuring a specific parameter within that context, and the feasibility of measuring the parameter within a specific context. Recommendations on off-line material characterizations for nanomaterial toxicity screening studies are presented in Table 2.

**Table 2:** Recommendations on material characterization

**Characterization (Off-line)**	**Human exposure**	**Toxicity Screening Studies**		
		
		**Supplied material**	**Administered material**	**Material *in vivo*/*in vitro***
Size distribution (primary particles)	E (Combine with agglomeration state)	E	D	D
Shape	E	E	O	O
Surface area	D	E	D	O
Composition	E	E	O	O
Surface chemistry	D	E	D	D/O
Surface contamination	D	N	D	N
Surface charge – suspension/solution	O	E	E	O
Surface charge – powder (use bio fluid surrogate)	O	E	N	O
Crystal structure	O	E	O	O
Particle physicochemical structure	E	E	D	D
Agglomeration state	E	N	E	D
Porosity	D	D	N	N
Method of production	E	E	--	--
Preparation process	--	--	E	--
Heterogeneity	D	E	E	D
Prior storage of material	E	E	E	--
Concentration	E	--	E	D

E: These characterizations are considered to be essential.

D: These characterizations are considered to provide valuable information, but are not recommended as essential due to constraints associated with complexity, cost and availability.

O: These characterizations are considered to provide valuable but non-essential information.

N: These characterizations are not considered to be of significant value to screening studies.

In addition, recommendations have been made on recording information on nanomaterial production, preparation, storage, heterogeneity, and agglomeration state. To enable retrospective interpretation of toxicity data and replication of tests, it is strongly recommended that all information on the production and processing of nanomaterials be recorded. Fully documenting storage time and conditions (including temperature, humidity, exposure to light and atmosphere composition) is essential, as physicochemical changes may take place over time. If possible, the physicochemical stability of samples over time should be demonstrated. Where a test material is a heterogeneous mixture of different components, information is required on the relative abundance of the different components, and whether associations in the bulk material are maintained in the administered material, or whether different components are preferentially administered with specific delivery mechanisms.

The agglomeration state of a nanomaterial during and following administration may have a significant impact on its biological activity. Agglomeration state at different structure scales should be characterized, including primary (primary particles), secondary (primary particle agglomerates and self-assembled structures) and tertiary (assemblies of secondary structures) scales. Ideally, agglomeration state in the biological environment following administration should be evaluated. If possible, some insight into the binding forces within agglomerates (e.g. relatively weak van der Waals forces or relatively strong sintered bonds) should be obtained. Material agglomeration or de-agglomeration in different liquid media should also be investigated where possible.

Characterization of material as administered is recommended as the highest priority, supplemented by characterization after *in vitro *or *in vivo *administration where possible, and followed in order of preference by characterization of the material as produced or supplied. Recommended characterizations in Table 2 reflect both this hierarchy and the feasibility of making measurements within the respective contexts.

#### 4.1.6 Analysis Methods

Many analytical techniques, both established and developmental, are available for characterizing the nanomaterial properties listed in Table 2. Table 3 lists some of the more widely available techniques and relates them to the nanomaterial characteristics of interest to toxicity screening studies. Techniques have been categorized with respect to their applicability to specific material characteristics. In general, the table is self-explanatory, and further information on each technique can be obtained from a wide range of sources. A number of techniques are only suitable for materials in certain forms, or specific classes of materials. For instance, while Transmission Electron Microscopy is capable of providing a wealth of information on nanoparticles and is considered a gold standard for evaluating particle size distribution and shape, dry (or in the case of cryo-TEM, frozen liquid-encapsulated) well-dispersed samples that are sufficiently robust to withstand high vacuums are required. Similarly, techniques such as Infrared (IR) spectroscopy are particularly sensitive to surface organic compounds, but are less useful for quantifying inorganic surface chemistry. In a number of cases, a complex technique such as TEM can be used to validate a characterization method that is more practical to use on a routine basis.

**Table 3:** Applicability of a range of analytical techniques to providing specific physicochemical information on engineered nanomaterials, in the context of toxicity screening studies

	**Analytical technique**															
		Transmission Electron Microscopy (TEM)	Scanning Electron Microscopy (SEM)	X-Ray Diffraction (XRD)	X-ray Photon Spectroscopy (XPS)	Auger Spectroscopy (AES)	Secondary Ion Mass Spectrometry (SIMS)	Scanning Probe Microscopy	Dynamic Light Scattering (DLS)	Zeta potential	Size Exclusion Chromatography	Analytical Ultracentrifugation	Differential Mobility Analysis (DMA)	Isothermal Adsorption (e.g. BET)	Spectroscopic techniques (UV vis, IR, Raman, NMR)	Elemental analysis (eg ICP-MS/AA etc)

**Physicochemical Characteristic**	Size distribution (primary particles)	▲	●	●				●	●		●	●	●			
	Shape	▲	●					●								
	Surface area	●	◇					◇	◇			◇		●		
	Composition	●	●	●			▲								●	▲
	Surface chemistry	●			●	●				◇						
	Surface contamination				●	●										
	Surface charge – suspension/solution									▲						
	Surface charge – powder (use bio fluid surrogate)									▲						
	Crystal structure	●	◇	▲												
	Particle physicochemical structure	▲	●													
	Agglomeration state	▲	●					●					●			
	Porosity	◇											●	●		
	Heterogeneity	▲	●					◇								

Other applicable techniques are available that have not been listed.

▲Highly applicable

● Capable of providing information in some cases

Capable of providing information in some cases, with validation from more accurate/applicable techniques

◇ Capable of providing qualitative or semi-quantitative information

Given the wide range of analytical techniques available in many disciplines associated with nanotechnology, multidisciplinary collaborations with research and analysis groups offering state of the art nanomaterial characterization capabilities are strongly recommended when carrying out nanomaterial toxicity screening studies.

#### 4.1.7 Research Gaps

1. The development of viable *in vivo *nanomaterial (including nanoparticles) detection techniques.

2. The development and production of inexpensive real-time monitoring instruments and methods for aerosol mass concentration (low concentrations, nanoscale particles), surface area concentration and size distribution.

3. The development of standardized, well characterized nanomaterial samples.

4. The development of radio-labeled nanomaterial samples, and samples that can be tracked and detected through neutron-activation.

5. The development of more advanced surface chemistry characterization techniques, in particular techniques capable of detecting and speciating biological molecules on the surface of nanoparticles and nanomaterials.

6. The development of electron microscopy techniques for biologically-relevant nanoscale analysis.

#### 4.1.8 Recommendations

1. All nanomaterial physicochemical characteristics that are potentially significant should be measured or be derivable in toxicity screening tests.

2. Characterization of nanomaterial as administered is strongly recommended, supplemented by characterization following administration where it is technically feasible and practicable. Characterization of the bulk material as-produced or supplied to the exclusion of the above is not recommended, except where more appropriate measurements are not feasible.

3. It is recommended that independent characterizations of nanomaterials (beyond information provided by producers and suppliers) are carried out where possible.

4. It is recommended that the following physicochemical properties of nanomaterials should be characterized in the context of toxicity screening tests: Particle size distribution, agglomeration state, particle shape, crystal structure, chemical composition (bulk and spatial), surface area, surface chemistry, surface charge, and porosity.

5. It is recommended that in all cases, sufficient information be collected to enable derivation of the delivered dose against all three primary physical metrics (number, surface area and mass concentration).

6. Off-line mass concentration measurements using filter-based methods are recommended as an essential component of inhalation nanomaterial screening tests. In addition, off-line measurement of aerosol size distribution is recommended.

7. On-line mass concentration and number measurements are recommended as an essential component of inhalation studies.

8. Multidisciplinary collaborations between research and analysis groups offering state of the art nanomaterial characterization capabilities are strongly recommended.

9. It is recommended that information on nanomaterial production, preparation, storage, heterogeneity and agglomeration state be recorded for all nanomaterial toxicity screening studies.

10. It is recommended that nanomaterial preparation methods are fully documented, including the selection of appropriate dispersion media, methods of dispersion in the medium and agglomeration state within the medium. Specific preparation techniques are not recommended, as these will depend on the material and test protocols being used. However, caution is advised when using ultrasonic agitation to disperse materials, as at high energies the method may be sufficiently aggressive to alter the material characteristics (see section 4.3.1.1).

### 4.2 *In vitro *Testing Methods

#### 4.2.1 Introduction

Before considering the application of specific *in vitro *testing methods to the assessment of the toxicity of nanomaterials, there are several generic issues that should be noted.

1) Advantages and disadvantages In general *in vitro *techniques are seen as an important adjunct to *in vivo *studies. These studies allow specific biological pathways to be tested under controlled conditions, as well as isolation of pathways that is not feasible *in vivo*; e.g., it is difficult to discriminate *in vivo *whether complement activation has a role in any pro-inflammatory effects of particles. The complement system can be isolated *in vitro*, and its potential role investigated. There are, of course, well-documented problems with *in vitro *approaches, including lack of validation against *in vivo *adverse effects, dosimetry mismatch, over-simplicity, non-involvement of the complete inflammatory response, etc.

2) Control particles It is important, in view of the above, that adequate positive and negative control particles are included in all experiments. This at least allows the test particle to be bench-marked against particles of known toxicity. These can include standard crystalline silica (quartz; e.g, Min-U-Sil or DQ12) as a known cytotoxic particle and fine TiO2 as an inert particle.

3) Expression of dose Toxicity and other responses should be expressed in relation to a range of dose metrics depending on the material and the dose metric data that are available (see Section 4.1).

4) Adsorption of proteins by nanoparticles The large surface area of nanoparticles means that they are capable of adsorbing proteins. Nanoparticles of various types have been reported to adsorb key proteins such as albumin [93], fibronectin and TGF-β [94]. This may confound endpoints that rely on the measurement of a protein as the protein may be produced but may also remove from the supernatant onto the nanoparticle surface by adsorption, providing a false-negative.

The *in vitro *tests that are presented will be divided into portal of entry toxicity and target organ toxicity. The potential target cells and associated appropriate endpoints will be described. Finally, research gaps and recommendations will be identified.

#### 4.2.2 Portals of Entry

##### 4.2.2.1 Lungs

The lungs represent a potential target for any airborne particles, and many *in vitro *models for the lung exist. Particles deposit on the airway or alveolar epithelium and encounter mucus or epithelial lining fluid. They may then interact with macrophages, which may result in their clearance, or they may enter the interstitium where they may make contact with fibroblasts and endothelial cells or cells of the immune system.

###### The Epithelium

The epithelium is the first barrier that confronts particles that deposit in either the conducting airways or the alveolar region. Therefore, both bronchial and alveolar epithelial cells should be considered as target cells for *in vitro *studies. Endpoints for detecting nanoparticle effects could include toxicity measurements, such as LDH release, for necrosis or various cytokine expression (IL-8, MCP-1 etc), [91,95] and activation of inflammation-related transcription factors such as NF-κB and AP-1[96,97]. Oxidative and nitrosative stress are dominant mechanistic hypotheses for cell damage and activation caused by pathogenic particles. These can be monitored by measuring oxidative stress using dichlorofluorescein [98] or oxidized glutathione as endpoints [99] and nitrosated proteins as a measure of active nitrogen species [100]. Responses to particle-induced oxidative/nitrosative stress can include up-regulation of anti-oxidant genes [101] such as superoxide dismutase and glutathione peroxidase, and so these can also be measured. Proliferative effects of nanoparticles can be assessed using a variety of assays including bromo-deoxyuridine incorporation [102].

If cancer is an endpoint that is under consideration, then direct measures of genotoxicity can be quantified by methods that include COMET assay and 8-hydroxy-deoxyguanosine measurement [103,104]. The translocation of nanoparticles across the epithelium could be an important discriminator of harmfulness and, although there are few publications specifically addressing transfer of particles across the epithelium *in vitro*, these should be developed and could contribute to understanding the factors that regulate translocation.

###### Macrophages

Macrophages play a key role in the cellular response to particles that deposit in the lungs. Macrophages could be affected by nanoparticles in various ways that can be studied *in vitro *through a variety of assays. Cellular cytotoxicity could be measured using conventional methods, such as lactate dehydrogenase release. Macrophage activation occurs following phagocytosis of a number of pathogenic particles leading to release of cytokines (tumour necrosis factor alpha (TNFα), interleukin-6 (IL-6) etc) and nuclear transfer of inflammation-related transcription factors nuclear factor kappa B (NF-κB) and activator protein 1(AP-1). Macrophages undergo an oxidative burst (OB) on phagocytosis of particles [105] and the extent of this in response to nanoparticles could be investigated. Nitric oxide (NO) may also be produced, in response to particles [106] and in the presence of superoxide radical peroxynitrite, a highly toxic species, can be produced [107]. If the OB or NO production is exaggerated, there could be 'bystander' injury to epithelial cells whilst diminished OB/NO production could mean impaired microbicidal activity that allows infection. Another key macrophage function reported to be impaired by nanoparticles is phagocytosis, [108] and so the effect of test nanoparticles on this function could be considered. The cytoskeleton is key to normal cell functioning and could be targeted by nanoparticles and so could be investigated.

###### Endothelial cells

Although these are found in the lungs, they are considered a part of the cardiovascular system and are dealt with below.

###### Fibroblasts

Fibroblasts are found in the interstitium and are liable to be affected by any particle that gains access to this site. At least two important modes of response could be activated by nanoparticle/fibroblast interactions and both modes constitute relevant endpoints for *in vitro *testing: 1) Pro-inflammatory effects, measured by cytokine/chemokine gene expression (TNFα; etc); or 2) fibrogenic responses activated either by direct stimulation of fibroblast growth or extra-cellular matrix secretion by the nanoparticle, or by autocrine stimulation following nanoparticle-stimulated release from the fibroblasts of growth factors such as transforming growth factor beta and platelet-derived growth factor.

###### The Immune System

Immunopathological effects could be envisaged if particles interact with lymphocytes, or as a consequence of their predilection for entering the interstitium, they modulate dendritic cell function. The effects of nanoparticles on immunological functions including antigen presentation by macrophages and dendritic cells and the subsequent effects on immune responses *in vitro *are relevant endpoints and appropriate tests should be designed.

###### Co-Cultures

In addition to monocultures of lung cells, co-cultures such as epithelial cells/macrophages or epithelial cells/endothelial cells may more closely represent the *in vivo *situation, and so such studies are encouraged.

###### Lung Slices

Methodology to culture whole lung tissue slices is available, such that multiple pulmonary cell types can be exposed *in vitro *in the same configuration as they occur *in vivo*.

###### Cell Lines vs. Freshly-Derived Cells

If possible, freshly-derived primary cells should be used. Where cell lines are used, these should preferably not be cancer cells. Where cancer cells are used, the endpoint response under study should be carefully compared to non-cancer cells to ensure that, for that endpoint, the fact that the cell is a cancer cell does not greatly modify the response compared to a non-cancer cell.

###### Whole Heart-Lung Preparation

The Langendorff heart-lung preparation may provide the opportunity to study the behavior of nanoparticles under highly controlled conditions. In this model the exsanguinated heart and lungs are maintained by perfusion and so transport between the lungs and the vascular space can be studied in the absence of blood [109].

##### 4.2.2.2 Skin

Skin or the integument is the largest organ of the body and is unique because it is a potential route for exposure to nanoparticles during their manufacture and also provides an environment within the avascular epidermis where particles could potentially lodge and not be susceptible to removal by phagocytosis [110]. What are the toxicological consequences of "dirty" nanoparticles (catalyst residue) becoming lodged in the epidermis? In fact, it is this relative biological isolation in the lipid domains of the epidermis that has allowed for the delivery of drugs to the skin using lipid nanoparticles and liposomes. Larger particles of zinc and titanium oxide used in topical skin-care products have been shown to be able to penetrate the stratum corneum barrier of rabbit skin with highest absorption occurring from water and oily vehicles [111]. This could also apply to manufactured nanoparticles. Can nanoparticles gain access to the epidermis after topical exposure, the first step in a toxicological reaction? Exposure to metallic nanoparticles, whose physical properties would allow them to catalyze a number of biomolecular interactions, potentially could produce adverse toxicological effects. More information is required regarding the efficiency of decontamination of nanoparticles from skin since solubilization and dilution, the two hallmarks of post-exposure decontamination, might be less efficacious for these solid structures.

Research should address the effects of dermal and systemic exposure to a number of types of nanoparticles in the skin. The skin is a primary route of potential exposure to toxicants, including novel nanoparticles. However, there is no information on whether particles are absorbed across the stratum corneum barrier or whether systemically administered particles can accumulate in dermal tissue. Nanoparticles may traverse through the stratum corneum layers at varying rates due to particle size or become sequestered within the epidermis to increase their exposure time to viable epidermal keratinocytes.

Nanomaterials are difficult to obtain in large quantities; therefore, it is best to conduct *in vitro *tests to estimate *in vivo *starting doses for toxicity testing [112]. At least three or four concentrations with controls should be used in all *in vitro *systems. These data would provide a preliminary, but relevant, assessment of both systemic *exposure *after topical administration as well as cutaneous *hazard *after both topical and systemic exposure, two essential components of any risk assessment.

###### Cell Culture

Human epidermal keratinocyte (HEK) monolayers can be affected by nanoparticle interactions. It has already been shown that changes in biomarkers of viability and toxicity can occur with exposure to multi-wall carbon nanotubes [50]. Cytotoxicity endpoints should be evaluated: 1) cell viability-metabolic markers such as mitochondrial reduction of tetrazolium salts into insoluble dye (MTT), 2) decreased cell viability-membrane markers like neutral red uptake into cell lysosomes, trypan blue exclusion and cell attachment/cell detachment, and 3) pro-inflammatory cytokine affects measured by TNFα, IL-8, IL-6, IL-10, or IL-1β. Genomics and proteomics assays could be used to explore the mechanism behind the toxicity. However, caution must be taken when using carbon black or any other material as a control because complications may occur. Carbon can adsorb the viability dyes, such as neutral red, and interfere with the absorption spectra. False positives will occur. The type of carbon black used is extremely important. For instance, ultrafine carbon black has been utilized in inhalation studies but dosing in cell culture gives different results, especially when conducting viability and cytokine assays.

Three dimensional skin cell cultures are also available commercially. They have shown to be able to predict irritation but may significantly overestimate absorption or penetration [113-116]. Assays listed above can be used but may not be applicable with nanomaterials due to adsorption.

###### Flow-through Diffusion Cell Studies

Diffusion cell system consists of flow-through diffusion blocks each containing multiple Teflon cells perfused by a constant temperature circulator through a Silastic oxygenator, an automatic fraction collector, and a desiccant. Circular fresh skin from pigs (pig skin mimics human skin and eliminates the extreme variability seen with random source human skin) or humans are placed epidermal – side up in Teflon flow-through diffusion cell. Compound containing nanoparticles is dosed on the epidermal side whilst the dermal side in each cell is bathed with receptor fluid at a set flow rate. The perfusate is collected at defined intervals up to 24 hrs and nanoparticles flux in the perfusate can be assessed by radioactivity counting, fluorescence, or UV detection. The skin surface can then be swabbed to remove non-absorbed surface particles and then tape stripped to remove a stratum corneum sample to assess nanoparticle penetration into this outermost epidermal layer. Serial sectioning of the skin can also be carried out [117,118].

###### Isolated Perfused Porcine Skin Flap (IPPSF)

The isolated perfused porcine skin flap (IPPSF) would be an ideal model to study the absorption and toxicity of nanomaterials. The IPPSF has an intact functional microcirculation, a viable epidermis and dermis and can be well controlled. A single-pedicle, axial pattern tubed skin flap is obtained from the abdomen of pigs following surgical creation of the flap perfused primarily by the caudal superficial epigastric artery and its associated paired venae commitantes. The IPPSF is transferred to the perfusion apparatus that is a custom designed temperature and humidity-regulated chamber. Nanomaterials can be topically dosed to the skin surface and perfusate samples collected over an eight hour period and assessed for nanoparticle flux [119-121].

Other acute toxicity *in vitro *assays are available but are used to test corrosives (rat transcutaneous electrical resistance (TER), commercially available EPISKIN, Epiderm and Corrositex) and irritation (EPISKIN, and Epiderm). However, the major traditional endpoint for skin toxicity is using the cell viability assay MTT reduction that has been shown to be unpredictable with nanomaterials due to marker interactions with nanoparticles.

##### 4.2.2.3 Mucosa

Mucosa is the moist tissue that lines particular organs and body cavities throughout the body, including the nasal cavity, oral cavity, lungs, vagina and gastrointestinal tract. Potentially one of the most important portals of entry for nanoparticle exposure (excluding the nasal cavity and lung, which has been detailed above) is the gastrointestinal tract. Either accidental or intentional exposure via oral administration to the GI tract can lead to significant exposures. Efficient uptake of nanoparticles via the GI tract has been well documented in oral feeding studies and gavage studies using particles ranging from 10 nm to 500 nm [122-124]. In these studies nanoparticles translocated through the mucosal lining and epithelial barrier of the intestine and were associated with the GALT (gastroinstetinal associated lymphatic tissue) and circulatory system within as little as 60 minutes time [125].

Intestinal epithelium can be studied using a variety of methods including immortalized cell-lines and tissue constructs. An example of immortalized cell-lines used to study the uptake of materials across the intestinal epithelial barrier include Caco cells, which have been used in many pharmaceutical studies to determine intestinal permeability [126].

These assays could be adapted for use in *in vitro *translocation rate studies or for developing a mechanistic understanding of the translocation process. IEC-6 and IEC-18 cell lines have been used extensively in mechanistic studies of the intestinal epithelial lining as well and may represent useful tools for nanoparticle research [127-129]. These cells have been used to measure the activation of various signal pathways after toxicant exposure, as well as cytokine and ROS/RNS release [130,131].

Dependent upon the specific application, vaginal and oral-lining exposure may be possible although it is unlikely that these, in general, would represent significant portal of entry exposure routes. However, there are a variety of cell-lines and tissue constructs or models available for study of translocation and impact of nanoparticle exposure through these routes [132-134].

#### 4.2.3 Cellular Assays

Study of target organs distal to the site of deposition pre-supposes that there is translocation and redistribution of nanoparticles away from the portals of entry in the lungs, skin or gut. As discussed above, potential target organs include blood, endothelium, neural tissue, heart, kidney, liver, and spleen.

##### 4.2.3.1 Endothelium

The endothelium is represented by a thin layer of cells lining the vasculature throughout the body. It has been demonstrated that ultrafine particles or nanoparticles may have a wide range of effects on the endothelium. *In vitro *cultures may be useful in elucidating mechanistic information about transport across the alveolo-capillary or blood-brain barrier and on endothelial cell effects. Cultured endothelial cells are well suited to determining the effects nanoparticles may have on RNS production which has been demonstrated to play a significant role in the homeostasis of the vasculature [135-137].

##### 4.2.3.2 Blood

*In vitro *studies using fractionated blood products (isolated red blood cells, platelets, leukocytes, or serum with complement) can be utilized in evaluating the effect on circulating blood. Activation of platelets, red blood cell interactions, production of ROS/RNS, cytokine/chemokine release from leukocytes, and complement activation are relevant endpoints to evaluate for nanoparticles. It has been demonstrated that nanoparticles have the ability to enter the circulatory system once translocation from site of entry has occurred [138,139].

##### 4.2.3.3 Spleen

The spleen is a major site of immune processing and lymphoid maturation, and accumulation of particles in the spleen may have consequences for immune responses and immunopathology. Spleen cells can be isolated and studied for the effects of nanoparticles *in vitro*. Endpoints could include antigen processing and immune responsivity *in vitro*, markers of lymphoid cell differentiation, and functional aspects such as dendritic cell function and lymphocyte proliferation.

##### 4.2.3.4 Liver

The liver is a complex organ and is structurally and functionally heterogeneous. The liver is the major site for biotransformation and defense against foreign materials and xenobiotics. It is an integral structure having two separate blood supplies, many different cell types, and many different functions. Liver injury, due to nanomaterials, may be characterized based on histologic lesions, such as inflammation or necrosis. Injury to the liver may also be characterized at the molecular level. Some of the most common mechanisms of hepatocellular injury are via the cytochrome P450 metabolic pathways. The liver can excrete materials into the bile; therefore, the biliary system may be exposed as well. *In vivo *there are reports that a variety of different toxins cause hepatocellular injury by a range of different mechanisms such as cytochrome P450 activation, alcohol dehydrogenase activation, membrane lipid peroxidation, protein synthesis inhibition, disruption of calcium homeostasis, and activation of pro-apoptotic receptor enzymes. Every effort should be made to use human derived cells for *in vitro *assays, because these studies could be used to predict toxicity in humans. However, there is a considerable human variability in enzyme function.

###### Primary Human Hepatocytes

Primary human hepatocyte cultures are available commercially with well-characterized metabolic profiles and a full complement of metabolizing enzymes. Availability of human cells is limited due to the increase in demand for liver transplantations.

###### Isolated Perfused Liver

This complicated model system would be suitable for the study of nanomaterials, because it is the closest model that mimics *in vivo *and would allow for a detailed characterization of particle distribution within the organ.

###### Liver Slices

Modern techniques of precision slicing have allowed liver slices to become a good model, because it retains the normal tissue organization which may be particularly critical for nanoparticle studies.

###### Collagen Sandwich Cultures

In this model system, the structural and functional integrity is retained for several days. The bile canaliculi are well preserved, and release of the enzymes alanine aminotransferase and aspartate aminotransferase can be evaluated.

In general, using these *in vitro *systems, hepatic metabolism can be studied using isolated hepatocytes and cell lines and evaluated for changes in CYP450. Microsomes may be used in screening nanomaterials for metabolism using LC/MS to identify metabolite formation. Subcellular fractions, liver slices and whole liver homogenates may be used to evaluate liver function and toxicity. To study the effects of nanomaterials on hepatic function specific endpoints, such as enzyme systems, mitochondrial function, albumin synthesis, cell detachment, gene and / or protein expression, and membrane damage should be considered. Mechanistically distinct endpoints could be utilized, such as cell morphology, viability, membrane damage, alamar blue metabolism, ATP content, covalent protein binding, peroxisomal proliferation, and GSH content. Other biomarker identifications, such as transcription and proteomic profiling should be studied. However, biomarkers may have limitations because immortalized cell lines are genotypically and phenotypically different from the organ itself. Also, hepatocyte cell cultures represent a single cell system and will only provide information on events that directly affect the cell itself. Some of these test systems may be able to predict the toxicity of nanomaterials as long as the assumptions and limitations are realized [140,141].

##### 4.2.3.5 Nervous System

###### Central Nervous System

*In vitro *systems to study the effects of particles on the nervous system could include culture of neurons and addition of nanoparticles to determine effects on neuronal function. Endpoints could include ROS/RNS production, apoptosis, metabolic status, effects on the action potential and ion regulation in general. Microglial cells are a type of macrophage found in the brain, and they may be involved in handling any nanoparticle that gets to the brain. The responses of microglial cells to nanoparticles should be studied along the lines of those described for macrophages in the lung section. Other cells that could be studied for effects of nanoparticles are astrocytes, glial cells that have a number of important roles that influence the behavior of neurons, and oligodendrocytes which provide support to axons by producing the myelin sheath, which insulates the axons.

###### Peripheral Nervous System

The skin and the other portals of entry and target organs will have a nerve supply and the skin for example, has sensory nerves that are present near the surface of the body. Nanoparticles have the potential to gain access to these nerves and be transported or affect them in a number of ways. This could be studied by using neuron culture of peripheral nerves and studying the effect of nanoparticles for various relevant endpoints (e.g., dorsal root ganglion neurons). In the autonomic nervous system both sympathetic and para-sympathetic neurons can be cultured, and effects of nanoparticles on their viability, metabolism, electrical activity and ionic homeostasis could be studied.

##### 4.2.3.6 Heart

Cardiac function could be altered by nanoparticles that find their way into the heart muscle from the microcirculation. Cardiomyocytes can be cultured and effects on their general viability, ionic homeostasis and metabolism could be ascertained. Additionally, cardiomyocytes beat with regular rhythm *in vitro*, and the effects on this could be measured and any effects examined as to mechanism.

##### 4.2.3.7 Kidney

The kidney is a major filtering system to eliminate toxicants from the bloodstream and it has been demonstrated that nanoparticles can be excreted via the kidney. Whether there are adverse effects of nanoparticles on the kidneys is unknown but this can be evaluated using *in vitro *techniques. Permeability assays used in pharmaceutical studies can be evaluated for use in measuring translocation and penetration through the renal tubules. Effects on the epithelial tubules and vasculature can be evaluated with existing cell culture techniques. A variety of endpoints can be evaluated, including signal transduction response, oxidative stress, cellular viability, ion channel flux, modulation in the release of growth factors and proteinases as important indicators to renal homeostasis. Several models exist that may be useful to investigate nanoparticle-kidney interactions including renal tissue slices to evaluate translocation, oxidative stress, signal transduction responses and toxicity [142,143]. Immortalized cell-lines are an inexpensive alternative to kidney slice models and may provide mechanistic information on the cytotoxicity of nanomaterials. Cells derived from isolated glomeruli, distal tubule/ collecting ducts, proximal tubule or proximal nephrons have been well characterized and are commercially available [144,145]. An example is the use of HEK-293 cells (human embryonic kidney cells) to evaluate cytotoxicity of chemicals [146]. MDKK cells and LLC-PK1 cells have been used extensively in *in vitro *mechanistic studies and can be utilized to evaluate effects of nanoparticles [147,148].

#### 4.2.4 Non-Cellular Assays

##### Durability

The ability of a particle to persist contributes to its ability to accumulate as dose. In fiber toxicology, there are well-documented dynamic and static protocols for assessing this property of durability *in vitro*, using Gambles balanced salt solution [149]. These fiber protocols could be modified to allow measurement of durability of nanoparticles *in vitro*.

##### Complement Activation

The complement system is a protein cascade that has evolved to detect foreign, mostly microbial, surfaces. It is, however, activated by asbestos [150] and by carbon nanoparticles [151]. Their high surface per unit mass and surface activity may mean that other types of nanoparticles might be potent at activating the complement system. This might modify the response by opsonising the particles (C3b) or causing inflammation by the production of anaphylatoxin (C5a). Studies on the ability of nanoparticles to activate the complement system are therefore warranted.

##### Adsorptive Properties

The large surface area of nanoparticles means that they can adsorb proteins [152,153]. Adsorption of different proteins might occur with different nanoparticle surfaces, and this could modify how they are handled by macrophages and other cells. This could therefore be a focus of study.

##### Free Radical Production

Most, probably all, pathogenic particles generate free radicals in cell-free systems, and this ability to cause oxidative stress contributes to their ability to initiate inflammation, and cause cell injury and genotoxicity [95,154-157]. The free radicals can arise as a consequence of stable radicals at the particulate surface (quartz) [158], redox cycling of ionic transition metal via the Fenton reaction (e.g. welding fume), [159] or by unknown surface mechanisms (nanoparticle carbon black) [160]. The ability of particles to generate free radicals can be assessed by a range of assays, including plasmid DNA scission [161], electron paramagnetic spin resonance, [162] and 8-OH-dG production in 'naked' DNA or a DCFD assay for *in vitro *ROS production [163].

##### Computational Toxicology

In addition to establishing screening methods mentioned above, efforts to assess risk associated with engineered nanomaterials or other environmental stressors should include a collection of new technologies called computational toxicology. Computational toxicology is defined as the application of mathematical and computer models and molecular biology approaches to improve prioritization of data requirements and risk assessments for environmental protection.

This approach involves four areas:

- computational chemistry which refers to physical-chemical-mathematical modeling at the molecular level and includes topics such as quantum chemistry, force fields, molecular mechanics, molecular simulations, molecular modeling, molecular design, and cheminformatics;

- molecular biology which allows for the characterization of genetic constituency and the application of wide coverage technologies, such as genomics, proteomics, and metabonomics, to provide the key indicators of cellular and organismal response to stressor input;

- computational biology or bioinformatics, which involves the development of molecular biology databases and the analysis of the data;

- systems biology which refers to the application of mathematical modeling and reasoning to the understanding of biological systems and the explanation of biological phenomena.

Computational toxicology is designed to increase the capacity to prioritize, screen, and evaluate materials by enhancing the ability to predict their toxicity. In addition to the "omics," quantitative structure-activity relationships (QSARs) developed in physical organic chemistry should be evaluated as to whether they can aid in predicting the structure-property relationship of nanomaterials. These multidisciplinary models could then be considered in a source-to-outcome continuum from environmental release through entire concentration, exposure concentration, target organelles, early biological effects, and adverse outcome.

While finding the relationship between structure and toxicity of nanomaterials using computational toxicology is likely a long time away, it is well to keep these models in mind while developing screening methods and to use current methods to validate and inform their development.

#### 4.2.5 Research Gaps

There is a paucity of data on the effects of nanoparticles on these different target cells and their respective endpoints *in vitro*. We therefore identify an urgent research need to obtain more information about nanoparticles in all of these systems. We do, however, identify some pressing needs and these include:

1. *In vitro *assays need to be used to determine important parameters that drive the toxicity and translocation potential of nanoparticles e.g. size, surface area, surface reactivity, etc.

2. Decisions have to be made regarding the most appropriate and useful *in vitro *endpoints and their relative utility and importance (e.g. translocation, generation of ROS, cytokine release, cytotoxicity). A ranking of these *in vitro *assays in order of relevance and utility should be attempted.

3. *In vitro *data should be used to develop a paradigm for nanoparticle toxicity that predicts the toxicity based on measurement of *in vitro *parameters; when mature, this paradigm could be critically tested *in vivo*.

4. Toxicokinetic data should be used to select the target cells and systems that are appropriate and to select plausible dose levels to use in the *in vitro *assays.

5. The effect of nanoparticle form (e.g. singlet particles or aggregates, use of surfactant) should be determined and the nanoparticle dose should be characterised as much as possible, e.g. regarding surface area, metals, etc.

6. There is a pressing need to prepare and choose appropriate benchmark materials for *in vitro *testing.

7. How do we interpret *in vitro *results without appropriate mechanistic information from *in vivo *models?

#### 4.2.6 Recommendations

Table 4 presents available *in vitro *systems for portal of entry testing. Table 5 presents available *in vitro *systems for other potential target organs.

**Table 4:** Available *in vitro *systems for portal of entry testing

**Portal of Entry**	**Cell/Tissue Type**	**Effect**	**Endpoint**	**Research Gap**
Lung	Epithelium	Toxicity	Trypan blue, LDH, apoptosis	
		Inflammation	Gene expression, oxidative stress, signal transduction pathways	
		Translocation	Transfer of nanoparticles across membranes	Translocation process
		Carcinogenesis	Genotoxicity, comet assay, 8OHdG, *hprt *assay, proliferation assay	
	Macrophages	Toxicity	Trypan blue, LDH, apoptosis	
		Chemotaxis	Chemotaxis assay	Recognition/Activation/Phagocytosis Process
		Phagocytosis	Particle uptake into cells, cytoskeletal staining	
		Inflammation	Gene expression, oxidative stress, signal transduction pathways	Additional markers?
	Immune Cells	Immune response	Cytokine profile, adjuvant effects	Additional markers?
	Endothelium	Inflammation	Adhesion molecules, oxidative stress	Additional markers?
		Coagulation	Von Willebrand factor, tissue factor	
	Fibroblasts	Inflammation	Oxidative stress, cytokine profile, gene expression profile	
		Fibrosis	Collagen synthesis, cell proliferation	
	Lung slices	Inflammation	Oxidative stress, signal transduction pathway, immuno-histopathology	
		Translocation	Particles across membranes	Translocation process
		Fibrosis	Collagen synthesis	
Skin	Cell systems (e.g. HEK)	Cytotoxicity Inflammation	Cell viability – MTT, neutral red, Cytokine profile	
	Flow-through diffusion systems	Absorption		
	Isolated Skin Flap Model	Absorption, Cytotoxicity, Inflammation	Glucose utilization, any other markers depending on end points (cytokine profiles, histopath, etc.)	
Mucosa	Intestinal epithelium (GI tract)	Cytotoxicity	Cell viability – MTT, neutral red, trypan blue Apoptosis	
		Inflammation	Cytokine profile, oxidative stress, signal transduction pathway	
		Translocation	Permeability assays	
	GALT	Inflammation	Cytokine profile, oxidative stress	
		Immune response	Adjuvant effects	Additional markers?
	Buccal epithelium (oral cavitiy)	Cytotoxicity	Cell viability – MTT, neutral red, trypan blue Apoptosis	
		Inflammation	Cytokine profile, oxidative stress, signal transduction pathway	
		Translocation	Permeability assays	
	Vaginal epithelium (reproductive system)	Cytotoxicity	Cell viability – MTT, neutral red, trypan blue Apoptosis	
		Inflammation	Cytokine profile, oxidative stress, signal transduction pathway	
		Translocation	Permeability assays	

**Table 5:** Available *in vitro *systems for potential target organs

**Target Organ**	**Cell/Tissue Type**	**Effect**	**Endpoint**	**Research Gap**
Endothelium	Endothelial cells (e.g. HUV-EC-C)	Cytotoxicity	Cell viability – MTT, neutral red, trypan blue Apoptosis	
		Homeostasis	Oxidative stress, gene expression profile	Additional markers?
		Translocation	Permeability assays	
Blood	Red blood cells, platelets, bone marrow (megakaryocytes)	Inflammation/Immune response	Platelet activation	
			Cytokine/chemokine release from leukocytes	
			Oxidative stress	
			Complement activation	
		RBC/particle interactions		Markers?
Liver	Hepatocytes	Toxicity	Cell viability – MTT, neutral red, trypan blue Apoptosis	
	Kupffer cells	Inflammation	Cytokine profile, oxidative stress, signal transduction pathway, gene expression	
		Coagulation	Von Willebrand factor, tissue factor	
	Isolated perfused liver slices	Translocation, distribution	Histopathology	Translocation process
	Liver slices	Toxicity studies	Cytotoxicity, P450 assay, ATP assays, GSH content	Additional markers? Genomics, Proteomics?
	Collagen sandwich cultures	Toxicity studies	Cytotoxicity, P450 assay, ATP assays, GSH content	Additional markers? Genomics, Proteomics?
Spleen	Lymphocytes	Immune response	Cytokine profile	
Central and peripheral nervous system	Neuronal cells	Toxicity	Cytotoxicity – Trypan blue, LDH Apoptosis	
		Inflammation	Cytokine profile, oxidative stress, signal transduction pathway, gene expression	
		Translocation	Gene expression, microscopic examination	
	Astroglial, Microglial cells	Inflammation	Cytokine profile, oxidative stress, signal transduction pathway, gene expression	
Heart	Cardiomyocytes	Toxicity	Cytotoxicity – Trypan blue, LDH Apoptosis	
		Inflammation	Cytokine profile, oxidative stress, signal transduction pathway, gene expression	
		Function	Beat – rhythm testing	
Kidney	Cell (e.g. HK-2, MDCK, LCC-PK1)	Toxicity	Cytotoxicity – Trypan blue, LDH Apoptosis	
		Inflammation	Cytokine profile, oxidative stress, signal transduction pathway, gene expression	
		Translocation	Permeability assays	Additional markers?
	Kidney slices	Toxicity	Cytotoxicity – Trypan blue, LDH Apoptosis	
		Inflammation	Cytokine profile, oxidative stress, signal transduction pathway, gene expression	
		Translocation	Permeability assays	Additional markers?

1. *In vitro *tests are recommended as they provide a rapid and relatively inexpensive way to assess the potential toxicity of nanoparticles; there are, however, well-documented drawbacks of the *in vitro *assays such as their relative simplicity and the high doses commonly utilized.

2. We recommend that "benchmark" particle controls be utilized in all studies such as crystalline silica and respirable TiO2.

3. Non-cellular tests including nanoparticle durability, complement activation, adsorption and free radical production can all yield valuable data on potential harmfulness of nanoparticles; computational toxicology may also make a contribution.

4. Attention should be given to the potential confounding effect of adsorption of proteins or assay constituents onto the nanoparticles surface.

5. Various cell-based systems are available with varying benefits and drawbacks, including single cell cultures of cell lines and freshly-derived cells, co-cultures, organ cultures (e.g. tracheal explants) and heart-lung preparations.

6. The lung is a key target organ and so lung epithelial cells, macrophages, immune cells and fibroblasts represent key cells for nanoparticle effects with specific regard to inflammation, immunopathology, fibrosis, genotoxicity, microbial defense and clearance.

7. Skin represents a target for nanoparticles, especially from nanoparticles in cosmetics and a number of in vitro test systems are recommended including keratinocyte culture, Flow-through Diffusion Cell, and Isolated Perfused Porcine Skin Flap (IPPSF).

8. Mucosa, the moist tissue that lines the nasal cavity, oral cavity, lungs, vagina and gastrointestinal tract also represents a potential target for nanoparticles and various *in vitro *systems are available for testing and should be utilized.

9. The tendency of nanoparticles to gain access to the vasculature means that endothelium and components of the blood are potential targets of nanoparticles and these can be studied *in vitro *and we urge that this pathway receive special attention.

10. The spleen, kidney, heart and liver will be target organs for bloodborne nanoparticles and we advise that a number of *in vitro *test systems are available to model effects in these organs.

11. Transfer of nanoparticles to the brain and interactions with the autonomic nervous system in the lungs have been reported and we strongly recommend that *in vitro *models be utilized to study the impact of nanoparticles in these important neural cells.

### 4.3 *In vivo *Assays

The following section details a two tier approach to *in vivo *assays. *In Vivo *assays are presented for pulmonary, oral, dermal and injection exposures. Tier 1 evaluations are presented for all routes of exposure and Tier 2 evaluations are presented for pulmonary exposures. Tier 1 Evaluations include markers of damage, oxidant stress, and cell proliferation. The Tier 2 evaluation for pulmonary exposures includes deposition, translocation, and biopersistence studies; effects of multiple exposures; potential effects on the reproductive system, placenta, and fetus; alternative animal models; and mechanistic studies. The section concludes with identification of research gaps and a summary of principal recommendations related to *in vivo *testing of nanomaterials.

#### 4.3.1 Pulmonary Exposure – Tier 1

Currently little information is available regarding airborne levels of nanomaterials generated during production and processing or quantities which may be aerosolized into the environment. However, due to their small size, aerosolization of respirable nanomaterials is likely, either as singlet or as aggregated particles and exposure by the inhalation route is a concern [48]. The following is a template which is recommended for the evaluation of possible adverse effects on the lung and other organ systems of pulmonary exposure to nanomaterials. A critical step in *in vivo *testing of nanomaterials is the characterization of the test material as described in Section 4.1.

##### 4.3.1.1 Exposure Method

###### Inhalation

Inhalation is the preferred method of exposure of the respiratory tract for hazard identification and to obtain dose-response data. Physicochemical characterization of the generated aerosol is essential. Of particular interest is information regarding the particle size distribution of the aerosolized nanomaterial, i.e., singlet nanoparticles vs. aggregates of primary particles. The generated aerosol must be well controlled for particle size and concentration, and attempts should be made to reproduce human exposure conditions for a specific airborne nanomaterial. A critical barrier to conducting inhalation studies with nanomaterials is that the amount of material is often limited. Intratracheal inhalation uses less material than whole body or nose-only inhalation exposures, but still requires more than may be available. Under such constraints, pulmonary exposure by intratracheal instillation, pharyngeal or laryngeal aspiration is acceptable for hazard identification. It has to be kept in mind that the upper respiratory tract will not be targeted.

###### Intratracheal Instillation

Intratracheal instillation of nanomaterial suspended in an appropriate vehicle is considered an acceptable method for pulmonary exposure to evaluate the relative toxicity of the test material [164]. Efforts should be made to disaggregate the nanomaterial suspended in vehicle. Nanoparticles vary significantly in their dispersibility; given the lack of other general techniques for disaggregating such particles, vortexing plus sonication is recommended. However, both probe and bath sonication may generate significant localized heat and pressure, disrupting surface coatings which were intentionally used to impart specific characteristic to the nanoparticles. Where sonication must be used, bath sonication is recommended. In all cases, the specific preparation technique (duration and power of sonication) should be reported. The characterization of the suspended particles should define the exposure material with respect to vehicle and degree of vortexing and sonication of the sample. Suspension of nanomaterial in a serum or surfactant-containing vehicle is sometimes employed to assist disaggregation of the nanoparticles. However, because these substances would adhere to the particle surface, the effect of such surface coatings on the biological activity of the particle is an issue which must be evaluated.

###### Pharyngeal and Laryngeal Aspiration

Pharyngeal aspiration has been shown to be an effective exposure method which results in a relatively even distribution of particles throughout the lungs [165]. A concern with the pharyngeal aspiration technique for pulmonary exposure is the unintentional aspiration of food particles from the oral cavity during this procedure. Therefore, food should be withheld the night before the aspiration exposure. Furthermore, a naïve group should be added in addition to the vehicle control to evaluate any pulmonary effects of aspiration alone [165]. As an alternative to pharyngeal aspiration exposure and to avoid contamination with materials from the oral cavity, laryngeal aspiration may be used, particularly in rats.

##### 4.3.1.2 Design

###### Animal Model

Currently, a large database exists using rats or mice to evaluate the pulmonary toxicity of particles. Therefore, for comparability to other toxicology studies, the use of the rat or mouse model is preferred. However, other animal models may be preferred to evaluate specific endpoints and would be acceptable.

###### Gender

At this time, there is no information concerning gender specific pulmonary sensitivity to nanomaterials. Therefore, there is no recommendation as to a preferred gender for these studies.

###### Dosimetry

Since mass may not be the proper dose metric for comparing the toxicity of fine vs. ultrafine particles [166,167], characterization of the test material should also include surface area per mass and particle number per mass. For practical purposes, dose could be monitored as mass delivered/animal or mass inhaled/animal and then be converted easily to a surface area or particle number dose as necessary, provided the correlation between these three particle parameters is available.

###### Benchmark Material

To place any pulmonary response to exposure to a given nanomaterial in perspective, results should be compared to those for particles of well-defined toxicity. Such benchmark materials could include nano-sized TiO_2_, carbon black, or crystalline silica. These benchmark materials should be characterized for surface area and particle number per mass, as well as for particle size and with respect to chemical purity and crystallinity to allow comparisons to be made using a variety of dose metrics.

###### Exposure Concentration

It is recommended that a minimum of three exposure levels be used. Information regarding the actual anticipated exposure levels in humans would be useful in determining the exposure concentration range to be evaluated. However, such information for nanoparticles is often lacking. In all cases, similar exposure concentrations of the test and benchmark materials should be used, and the various dose metrics discussed above should be considered when choosing the exposures for the benchmark and test materials. It is recommended that the highest concentration chosen should exhibit toxicity with the benchmark material.

###### Exposure Duration

For intratracheal instillation or pharyngeal aspiration, a single exposure to the nanomaterial is sufficient for Tier 1 studies. Caveat: consider high dose and bolus effect! For inhalation, a two week exposure is recommended, although shorter exposures, perhaps at higher concentrations, should be done if this mimics human exposures.

###### Pulmonary Parameters

Pulmonary responses should be monitored 24 hours to 28 days post-exposure. A suggested time course could be 24 hrs, 1 week and 28 days post-exposure.

##### 4.3.1.3 Pulmonary Endpoints

###### Inhalation Studies

The degree/intensity and duration of pulmonary inflammation and cytotoxic effects following nanoparticle exposures are important endpoints for assessing the toxicity of a test nanoparticle.

1. Bronchoalveolar lavage (BAL) damage markers – BAL profile. This method samples the cells and fluid from the bronchoalveolar space and allows the assessment of inflammation by quantification of cell numbers and types and components of the fluid phase. In addition, considerable extra information can be gained by various *ex vivo *manipulations of the BAL cells, e.g., gene expression, phagocytic potential, etc. Other BAL damage markers include BAL lactate dehydrogenase levels (as a measure of cytotoxicity), BAL protein levels (increases in BAL fluid protein concentrations generally are consistent with enhanced permeability of vascular proteins into the alveolar regions, indicating a breakdown in the integrity of the alveolar-capillary barrier), and BAL alkaline phosphatase levels (as a measure of Type 2 alveolar epithelial cell toxicity). Methodologies for cell counts, differentials, and pulmonary biomarkers in lavaged fluids have previously been described [168,169].

2. Oxidative stress markers – ROS/RNS. Reactive oxygen and nitrogen species have been implicated in DNA damage and induction of inflammatory cytokines and growth factors. Acellular BAL fluid levels of gluthathione, total antioxidants, or nitrate/nitrite (a measure of nitric oxide production), lipid peroxidation of lung tissue, or *ex vivo *measurement of ROS/RNS from BAL cells can be employed to monitor oxidant generation and oxidant stress. Methodologies for oxidative stress markers have been described [170,171].

3. Histopathology – Description of the general effects of treatments on the lungs should include endpoints such as presence of dust-laden macrophages, cellular infiltrates and hyperplastic changes in the epithelium. It is recommended that the entire respiratory tract be evaluated for adverse pathological effects. This would include the upper respiratory tract – the nose, larynx and upper airways; the lower respiratory tract and lymph nodes; and the pleural region. Histopathological observations in a Tier 1 process would focus primarily on inflammatory responses and the development of fibrosis. Fibrosis can be determined in lung tissue by specific staining of collagen in histopathological slides, or by qualitative and quantitative histopathology.

4. Cell proliferation – Increased cell division plays a key role in pathological responses and can be determined in epithelial or mesothelial cells by uptake of labeled nucleotide precursors, such as tritiated thymidine or BrdU. Recommended experiments are designed to measure the effects of particle exposures on airway and lung parenchymal cell turnover in rats following exposures. Groups of particulate-exposed rats and corresponding controls can either be pulsed or implanted subcutaneously with minipumps containing 5-bromo-2'deoxyuridine (BrdU) dissolved in a sodium bicarbonate buffer solution. Methodologies for cell proliferation studies have previously been described [168,169]. An alternative method to BrDU staining is the PCNA staining method. Proliferating cell nuclear antigen (PCNA) is a nuclear protein associated with cell proliferation and has been used to discriminate via immunohistochemistry proliferating cells in numerous tumor types including those found in the lung [172-174].

###### Intratracheal Instillation or Pharyngeal/Laryngeal Aspiration Studies

As discussed above, inhalation is the most physiologically relevant and therefore preferred method of pulmonary exposure for hazard identification and to obtain dose-response data. However, both intratracheal instillation of nanomaterials suspended in an appropriate vehicle and pharyngeal or laryngeal aspiration (with appropriate caveats) are considered to be acceptable methods for pulmonary exposure to evaluate the relative toxicity of the test material. Similar to studies using aerosol exposures, the following pulmonary endpoints should be evaluated in a Tier 1 testing strategy approach for assessing lung hazards to nanoparticles:

1. BAL damage markers

2. Oxidative stress markers

3. Histopathology

4. Cell proliferation

##### 4.3.1.4 Other Organ Endpoints

Exposure to nanoparticles *via *the respiratory tract includes a high probability of translocation to other organs and tissues – depending on nanoparticle size and surface chemistry. Although translocation rates may be very low, localization at sensitive sub-cellular sites (e.g., mitochondria) could result in adverse responses directly induced by the nanoparticle. Alternatively, or in addition, potential oxidative stress and inflammatory responses elicited by nanoparticles in the respiratory tract may result in the release of mediators which can lead to indirect secondary effects in extra-pulmonary organ systems. Thus, it is essential to include an evaluation of potential effects in remote organs and tissues, such as liver, spleen, bone marrow, heart, kidney, and CNS, in the Tier 1 evaluation.

Histopathological examination of extra-pulmonary tissues should be mandatory; however, this alone may only show significant effects following longer-term or very high exposures. Therefore, consideration should also be given to determining organ-specific endpoints, such as acute phase proteins and coagulation factors, for effects on the cardiovascular system, immune response assays for effects on the spleen, and immunohistochemical staining for dopaminergic neurons in brain sections to evaluate neurogenic effects. Additional functional assays (*e.g*., measurement of heart rate variability) may be considered, but since they require specific equipment and expertise, these are not mandatory for Tier 1 studies.

#### 4.3.2 Pulmonary Exposure – Tier 2

Research results showing that nanoparticles can translocate from the portal of entry, the respiratory tract, via different pathways to other organs/tissues makes them uniquely different from larger-sized particles in that they may induce direct adverse responses in remote organs. In particular, such responses may be initiated through the interaction of nanoparticles with sub-cellular structures following endocytosis by different target cells. For this reason, special attention needs to be given to recognizing such effects, which in the healthy organism are probably very subtle initially, even not detectable, but could have serious consequences in a compromised organism or a compromised organ. Examples are effects of anthropogenic ultrafine particles in asthmatics, in people with cardiovascular diseases, in the elderly and very young. Complementary Tier 2 studies can be used to obtain more data for hazard identification as an initial step for risk assessment. Tier 2 studies will provide additional information to either characterize further effects seen in Tier 1 studies or to obtain new data using specific models of susceptibility. Ideally, studies should be performed using inhalation exposures as a first choice, in particular if a positive response was seen in Tier 1 studies when intratracheal instillation or pharyngeal/laryngeal aspiration was used. If insufficient amounts of material are available, multiple low dose (1–10 μg/kg body weight) exposures of the respiratory tract using the above-mentioned non-inhalation methods can be applied, *i.e*., dosing once or twice/week for 4 weeks with 2–3 months follow-up.

##### 4.3.2.1 Animal Models

The absence of an effect of nanoparticles in a normal animal model does not imply that there will be no effect in a model which exhibits enhanced susceptibility. Increased susceptibility can be due to a number of factors, including age, disease, altered organ function, genetic polymorphism. Respective animal models include exposures of senescent, transgenic and knockout animals and animals with compromised organ systems (*e.g*., hypertension; diabetes models; immuno-compromised, infectivity models). In general, susceptibility models include compromised functions of the respiratory tract, CNS, cardiovascular system (dysfunction of endothelial cells, platelets), bone marrow, and/or kidney. It is essential that the models used are relevant to the human disease state and that a respective animal model has been validated in the peer-reviewed literature. In addition to obtaining information for identifying nanoparticle hazards, Tier 2 studies will also provide data on underlying mechanisms which can be used in concert with the mechanistic *in vitro *studies.

##### 4.3.2.2 Multiple Exposures

As indicated above and under Tier 1 studies, repeated inhalation exposures are the first choice for realistic dosing. Information about the physicochemical nature of airborne nanoparticles at the workplace, or anticipated exposure of the general public (consumer), is essential to mimic the same for animal exposures. Issues include: are the nanoparticles aggregated or singlets; what is the diameter in the airborne state; what is known about other chemical characteristics (see discussion in this document, Section 4.1.)? Generation and monitoring of airborne nanoparticles for inhalation exposures requires special equipment and expertise. As an alternative to inhalation, intratracheal instillation, oropharyngeal or laryngeal aspiration can be used. However, unless the nanoparticles are coated and made "soluble" in physiological solutions, artifacts due to aggregation may occur when using these non-inhalation methods. In addition, the upper respiratory tract is circumvented by the non-inhalation methods, thereby eliminating potential neuronal nanoparticle translocation to the CNS. To address this concern, one could expose the animal to nanoparticles by nasal instillation. Techniques for tracking the uptake of nanoparticles by sensory neurons would be similar to those discussed in the section concerning deposition, translocation, and biopersistence (Section 4.3.2.3). Furthermore, the impact of coating for purposes of "solubilizing" the nanoparticles has to be carefully considered in cases where anticipated human exposure is to the uncoated material, since cellular uptake, translocation and effects will be affected by the surface coating.

Daily repeated inhalation exposures over four weeks are suggested for the Tier 2 studies, with an up to 3-month post-exposure observation period, including interim post-exposure sacrifice days. With respect to the non-inhalation methods of exposure, dosing can occur 1–2 times/week over a 4-week period, followed by the post-exposure observation period. Special attention needs to be given to the selection of exposure concentrations (inhalation) and doses (instillation, aspiration). Knowledge about anticipated human exposure will be extremely valuable, and use of predictive particle deposition models (*e.g*., MPPD model) can be used to determine realistic exposure concentrations/doses.

The Tier 2 studies are aimed at obtaining additional information with respect to the biokinetics of the nanomaterials following exposure of the respiratory tract (see below, "Deposition, translocation and biopersistence studies"), the stability of nanoparticles in the organ system (*e.g*., *in vivo *change of surface chemistry, bioavailability of core material), and the potential acute and sub-acute effects in the mammalian organism, including genomic and proteomic evaluation. Other endpoints are the same as listed under Tier 1 studies for the respiratory tract.

##### 4.3.2.3 Deposition, Translocation and Biopersistence Studies

Exposure to airborne nanoparticles *via *the inhalation route leads to deposition in the various compartments of the respiratory tract according to probabilities dependent on three important parameter groups: aerodynamic and thermodynamic nanoparticle properties, breathing pattern, and the three-dimensional geometry and structure of the respiratory tract. Deposition probability of nanoparticles below a thermodynamic diameter of 500 nm increases with decreasing size because of the increasing diffusion velocity leading to an increased deposition in the small airways and the alveoli, in particular. Below 20 nm, the location of deposition of nanoparticles changes to the upper respiratory tract because of their even higher diffusion velocity [175]. Currently existing computer codes provide a first estimate of deposition probabilities in the various regions of the respiratory tract which may require modification if there are indications that nanoparticles may undergo changes of their aero- and/or thermodynamic properties not being considered in those codes [176,177].

Once deposited, insoluble nanoparticles undergo clearance mechanisms specific to the region of the respiratory tract; *i.e*., at all regions nanoparticles will interact with proteins of the epithelial lining fluid potentially forming complexes which are likely to affect their subsequent metabolic fate and biokinetics [178]. On the epithelium of conducting airways, mucociliary clearance provides a rapid transport, to the larynx for further transport into the gastro-intestinal tract and excretion. Note, however, that there also occurs long-term retention in human airways for a fraction of nanoparticles which increases with the decreasing size of nanoparticles giving rise to cellular uptake. On the epithelium of the alveolar region there is no rapid transport so that phagocytosis by free phagocytes can occur with subsequent slow clearance to the larynx, but because of the limited capability of macrophages to recognize nanoparticles, endocytotic processes and trans-cellular transport by other cells like epithelial type I + II cells, become prominent together with para-cellular transport mechanisms across tight junctions under inflamed conditions [179,180]. These mechanisms result in translocation of nanoparticles into the interstitium, lymphatic drainage and possible translocation across endothelial cells into capillaries. This access to the blood circulation provides accumulation and possible adverse reactions in secondary target organs such as the cardiovascular system, liver, spleen, bone marrow, central nervous system, endocrine organs and interaction with endothelial cells, platelets and immuno-competent cells in the circulation. Besides the direct effects of translocated nanoparticles on secondary organs, indirect effects may occur as well triggered by interactions of nanoparticles at their site of retention in the respiratory tract with adjacent biological systems like cells, fluids, proteins and extracellular matrix. Subsequent cell activation can lead to release of cytokines and other mediators which subsequently diffuse into the circulation to induce adverse responses in secondary target organs. Because the underlying mechanisms of nanoparticle translocation and accumulation or mediator response in secondary target organs are not fully understood, the determination of nanoparticle kinetics should be a high priority.

The scenario described above relates to biopersistent nanoparticles which maintain their particulate state; however, nanoparticles even when they may be insoluble in water may not persist as a solid particle in cells and body fluids but may fully or partially (e.g., surface coating) disintegrate/dissolve, so that eventually a biopersistent core with different particle properties/toxicities is retained. Because of the high diversity of emerging nanoparticles, studies of their biopersistence should be regarded of high priority.

Methodologies for such biopersistence studies include:

• *Radio-labeled or fluorescently or magnetically tagged monitoring in lungs and various organs*

Radio-labeling of nanoparticles specifically with a radio-isotope of one of the contextual chemical elements of the particle matrix is the gold standard for studying translocation kinetics allowing for time-efficient, extremely high sensitivity and specificity measurements, particularly when they aim to account for a balance of the entire distribution in the body and in excretions [79,181]. Also fluorescent or magnetic labeling provides a powerful means of high sensitivity and specificity to determine translocated fractions in various target organs [181,182]. Extreme care should be taken; however, to assure that any label stays firmly with the nanoparticles, otherwise the results will be severely flawed. Also, evidence is required that the process of labeling does not modify the nanoparticle in its function and its surface since surface is the predominant interacting substrate with biological systems.

• *If impossible to tag: EM to monitor deposition and fate*

Labeling of nanoparticles will not be possible in every case. In this event tracking of nanoparticles by electron microscopy may be a suitable alternative to monitor the fate of electron-dense nanoparticles in the organism. The method of choice for evaluation is a quantitative morphometric approach, which is by far preferable over qualitative spotting images, which may lead to a wrong interpretation. In addition, it is strongly recommended to search for adequate alternate labeling techniques, which may not be obvious at the first glance but would still provide a feasible option, for example use of confocal microscopy with fluorescently labeled nanoparticles.

• *Chemical analysis may be possible*

If the matrix of the nanoparticle provides a characteristic chemical element or compound, chemical analysis of this characteristic provides another strong alternative to track the fate of nanoparticles. Importantly, contamination and possible endogenous background levels require careful distinction as well as the determination of the lower level of sensitivity of the analytical approach.

##### 4.3.2.4 Genomics and Proteomics

Nanomaterials distributing to different tissues of the body following deposition in the respiratory tract can potentially affect multiple cellular functions, and it will be difficult to determine with conventional assays what changes and adverse effects may have occurred. Use of genomic and proteomic analyses should be considered, the former providing information about specific mechanisms at the molecular level (*e.g*., oxidative stress) and the latter linking this to the expression of proteins resulting in effects at the cellular and tissue level. Results from such analyses are needed to help in the interpretation of responses. Analysis of the results of these assays requires the input of bioinformatics which will help in the interpretation of elicited responses. Together, genomic and proteomic studies represent an effective strategy combining hypothesis-forming and hypothesis-driven research which is needed in the assessment of nanoparticle risks within the framework of a multidisciplinary team approach.

##### 4.3.2.5 Effects on the Reproductive system, Placenta, and Fetus

The studies to assess Tier 2 reproductive effects following pulmonary exposures to nanoparticles should follow protocols similar to the OECD Guideline 422 for Testing of Chemicals (Combined Repeated Dose Toxicity Study with the Reproduction/ Developmental Toxicity Screening Test – adopted 3-22-1996). The test substance should be administered in gradual doses to several groups of male and female rats. Males should be dosed for a minimum of 4 weeks (which includes a minimum of 2 weeks prior to mating during the mating period and approximately 2 weeks post mating). Given the limited pre-mating dosing period in males, fertility may not be a particularly sensitive indicator of testicular toxicity and should be concomitant with a detailed histopathological analysis of the male gonads to assess impact on fertility and spermatogenesis.

Females should be dosed throughout the study – including 2 weeks prior to mating (with the objective of covering a minimum of 2 estrus cycles), the variable time to conception, the duration of pregnancy, and a minimum of 4 days after delivery, up to and including the day before the scheduled sacrifice. The duration of gestation should be recorded and is calculated from day 0 of pregnancy. Each litter should be examined as soon as possible after delivery to establish the number and sex of pups, stillbirths, live births, runts (pups that are significantly smaller than corresponding control pups), and the presence of gross abnormalities.

Live pups should be counted and sexed and litters weighed within 24 hours of parturition (day 0 or 1 post-partum) and on day 4 post-partum. In addition to the observations on parent animals, any abnormal behavior of the offspring should be recorded.

#### 4.3.3 Oral Exposure – Tier 1

It is possible that during the life of a nanomaterial (production, application, disposal, etc) it may appear in the water supply or be inadvertently ingested. If this is a concern, the effects of oral exposure to the nanomaterial should be investigated. Exposure should be by a single gavage at a dose which would represent the worse case human exposure. As with pulmonary exposure, for oral exposure the physical and chemical properties of the test material should be characterized in the form delivered to the test animal. Rats or mice are the recommended model system. There is no preference concerning gender for such studies. The feces should be collected for four days post-exposure, and the amount of nanomaterial eliminated vs. retained should be determined. Particularly GALT, mesenteric lymph nodes and liver should be analyzed for the presence of nanoparticles. If absorption of the nanomaterial from the gastrointestinal tract is near zero, then the systemic effects of oral exposure to that nanomaterial need not be evaluated. However, if significant absorption of the nanomaterial is evident, evaluation of systemic toxicity is recommended using histology and functional assays as described for the various organ systems after pulmonary exposure.

#### 4.3.4 Injection – Tier 1

Some nanomaterials are being evaluated as drug delivery systems. In such a case, the potential toxicity of this nanomaterial after injection should be evaluated. Rats or mice are the recommended model system. There is no preference concerning gender for these studies. If possible, a tagged nanoparticle should be injected and its distribution to various organs (liver, spleen, heart, bone marrow, kidney, and lung) and elimination in the feces should be monitored for a week post-exposure. Histology and functional assays (e.g., mitochondrial function) of the various organ systems should be implemented as described following pulmonary exposure (Section 4.3.1.4).

#### 4.3.5 Skin Exposure – Tier 1

For skin absorption of nanomaterials, the most appropriate animal model should be used. Rats are most common but rabbits, guinea pigs and pigs are also used to assess toxicity and irritation. The rat and pig are recommended as the animal of choice. The rat being small and already having an established database in the field of toxicology by other routes of exposure could be used because the amount of nanomaterials needed would be less for this small species. Frequently, the domestic pig is utilized in absorption studies because the skin is anatomically, physiologically and biochemically similar to that of humans. Twenty-four hours prior to dosing with nanomaterials, the area (10% of the body surface) on the back should be clipped to remove hair. Three doses at log intervals (mg/cm^2^) plus controls (vehicle, no material controls and a positive control) should be applied to both normal skin and abraded skin to mimic how humans are exposed. The material should be applied in an occluded fashion because nanomaterials, unlike many chemicals, will not be absorbed into the skin immediately. The occlusion device (site protection) used to prevent the nanomaterial from falling off should be attached to the surface of the skin by non-irritating tape. At least 4–6 animals per group/ dose plus controls and vehicle controls should be utilized over the duration of 24 hrs. At 0.5, 1, 2, 4, 8, and 24 hrs, the treatment sites should be scored for erythema and edema using the Draize test scores. Skin biopsies should be taken for transmission electron microscopy that would identify cellular changes as well as localize the penetrated particles within the skin. Light microscopy could be utilized to assess the morphological alterations that could occur due to the acute toxicity of the nanomaterials but this will not detect nanomaterial localization. For repeated exposures, nanomaterials should be applied daily for 5 or 7 days and could continue until 28 days. This could depend on the type and the amount of nanomaterials that are available. If a 28 day study is planned, then daily clinical observations should be conducted. At termination, hematology, clinical chemistry, evaluation of local lymph nodes and an immunotoxicology battery of tests should be performed. Standard full necropsy exam (liver, kidney, etc) should also be conducted [183,184]. The specific goal of the study, dermal absorption or irritation, will dictate the specific study design (e.g. duration of study, samples collected).

#### 4.3.6 Research Gaps

Significant research gaps exist; the first four included in the following list pertain to general informational needs on production, use, and exposure to nanomaterials that would be helpful in the design of toxicity test.

1. What is being made and in what quantities in the nanotechnology industry?

2. What exposure levels are likely in the workplace?

3. What is/are the likely route(s) of exposure?

4. What are occupational vs. environmental exposures?

5. Radio-labeled particles are needed for investigation of deposition, translocation, and biopersistence. This requires specific lab that can work with and detect labeled materials; labeling not feasible for many materials.

6. A source of reference nanomaterials should be available to researchers.

#### 4.3.7 Recommendations

1. Studies involving *in vitro *(non-cellular and cellular), pulmonary, oral, injection and dermal exposure are recommended in Tier 1 testing.

2. It is essential that exposures in animal models be relevant to human exposures, when known, for production, use, and disposal.

3. It is recommended that exposure route be relevant to anticipated human exposures for production, use, and disposal.

4. It is recommended that the test material be fully characterized, preferably as delivered to the animal.

5. Following pulmonary exposure, recommended endpoints to be measured include organ-specific markers of inflammation, oxidant stress and cell proliferation (*e.g*., mitochondrial) and histopathology in the lung as well as measurement of damage to non-pulmonary organs.

6. Studies involving a) use of susceptible models, b) multiple exposures, c) evaluation of deposition, translocation and biopersistence, d) reproductive effects, and e) mechanistic genomic and proteomic techniques may be considered for Tier 2 testing.

## 5.0 Conclusion

Engineered nanomaterials presenting a potential risk to human health include those capable of entering the body and exhibiting a biological activity that is associated with their nanostructure. Nanomaterial-based products such as nanocomposites, surface coatings and electronic circuits are unlikely to present a direct risk as exposure potential will be low to negligible. Nanomaterials that are most likely to present a health risk are nanoparticles, agglomerates of nanoparticles, and particles of nanostructured material (where the nanostructure determines behavior). In each of these cases, exposure potential exists for materials in air and in liquid suspensions or slurries.

Recognizing the early stage of understanding of the potential toxicity of nanomaterials and that little knowledge exists regarding specific nanomaterial characteristics which may be indicators of toxicity, the elements of a screening strategy outlined in this document include a significant research component. The range and extent of recommended testing reflect this developing state of knowledge. The elements of a screening strategy are clear, but the detailed approach will evolve and become more focused and selective as the results of these early-stage screening/research studies become available. A more thorough discussion of the 'elements' presented and the development of a more robust and detailed strategy will only be possible as knowledge increases. Elements of a nanotoxicity testing strategy which have been detailed in previous sections are summarized below.

### Physicochemical Characterization

Appropriate physicochemical characterization of nanomaterials used in toxicity screening tests is essential, if data are to be interpreted in relation to the material properties, inter-comparisons between different studies carried out, and conclusions drawn regarding hazard. The dependence of nanomaterial behavior on physical and chemical properties places stringent requirements on physicochemical characterization and includes assessing a range of properties, including particle size distribution, agglomeration state, shape, crystal structure, chemical composition, surface area, surface chemistry, surface charge and porosity. Precise requirements will differ for *in vivo *and *in vitro *studies, and according to the material delivery route or method. In addition, characterizing human exposures introduces a third set of requirements.

A wide range of analytical methods are available that are applicable to nanomaterials, and multidisciplinary collaborations are encouraged to ensure appropriate methods are adopted. Particular consideration should be given to the use of Transmission Electron Microscopy, which in many cases can be considered the gold standard of nanoparticle characterization. In addition, information on nanomaterial production, preparation, storage, heterogeneity and agglomeration state should be recorded in all cases. Characterization of nanomaterials after administration *in vitro *or *in vivo *is considered the ideal in screening studies, although it currently presents significant analytical challenges. Characterization of the material as administered is therefore recommended for most screening tests. Characterization of the nanomaterial solely as produced or supplied is only considered appropriate where the previous two approaches are not viable. In all screening studies, dose should be evaluated against appropriate metrics. The three principal physical metrics of interest are mass, surface area and number concentration of particles: given current uncertainty over the relevance of each, it is important that all three are measured or derivable in any given study.

### *In Vitro *Testing Methods

*In vitro *tests of toxicity yield data rapidly and can provide important insights and confirmations of the mechanism of *in vivo *effects. We recommend that a wide range of *in vitro *tests be applied to the key research questions relating to the potential hazard associated with nanoparticles exposure. A wide range of *in vitro *approaches exist that can be matched to specific questions relating to different aspects of nanoparticles toxicity. Non-cellular tests can provide information on aspects such as biopersistence, free radical generation by particle surfaces and activation of humoral systems such as the complement system; computational toxicology methods may also be useful. Cell-based systems can comprise cell lines and freshly derived primary cells in monocultures or co-cultures. Organ cultures and heart/lung preparations are also potentially useful for studying nanoparticles effects and translocation. We recommend that the *in vitro *tests should reflect the different portals of entry and target organs that nanoparticles could impact and these include lung, skin, mucosal membranes, endothelium, blood, spleen, liver, nervous system, and heart. As always, care should be taken in interpreting data obtained from *in vitro *systems because of the high doses normally used *in vitro *and the impact of a bolus effect. There should be inclusion of appropriate benchmark particles to contextualize the results of *in vitro *assays. We recommend vigilance for artifactual effects peculiar to nanoparticles caused by their large adsorptive surface which can deplete cell products or assay constituents and thereby confound assay results. In addition to the utilization of existing test systems we suggest that new assays may be developed, for example to study transit of nanoparticles across cell layers.

### *In Vivo *Testing Methods

For *in vivo *testing of nanomaterials, two tiers of studies are discussed. Tier 1 studies would involve pulmonary, oral, injection, and dermal exposure as would be relevant to the human exposure(s) of concern. A critical initial step in *in vivo *testing is full characterization of the test material. Endpoints of concern for pulmonary exposure involve organic-specific markers of inflammation, oxidant stress, and cell proliferation and histopathology in the lung as well as measurement of damage to non-pulmonary organs. Tier 2 pulmonary exposure studies are recommended but not mandated. These studies would provide useful information for a complete risk assessment of a nanomaterial. Tier 2 studies include: 1) use of susceptible models, 2) effects of multiple exposures, 3) deposition, translocation and biopersistence studies, 4) evaluation of reproductive effects, and 5) mechanistic studies employing genomic and proteomic techniques.

The testing strategy for *in vivo *studies would be of greatest value for hazard identification and risk assessment if exposure dose, route of exposure, and particle characteristics closely modeled those of human exposure. Therefore, an understanding of the life cycle of a given nanomaterial, i.e., exposure during production, upon use, and environmentally, is a critical research need.

## 6.0 Competing interests

The author(s) declare that they have no competing interests.

## 7.0 Authors' contributions

Günter Oberdörster served as the Chair of the Nanomaterial Toxicity Screening Working Group. Andrew Maynard served as the Chair of the physiochemical characteristics sub-group, Ken Donaldson served as the Chair of the *in vitro *testing methods sub-group, and Vincent Castranova served as the Chair of the *in vivo *assays sub-group. Julie Fitzpatrick managed this project for the ILSI Research Foundation/Risk Science Institute (Table 6). 

**Table 6:** 

The Working Group members are listed below:	
Günter Oberdörster (Chair)	University of Rochester
Kevin Ausman	Rice University
Janet Carter	Procter & Gamble Company
Vincent Castranova	National Institute for Occupational Safety & Health
Ken Donaldson	University of Edinburgh (UK)
Julie Fitzpatrick	ILSI Research Foundation/Risk Science Institute
Barbara Karn	U.S. Environmental Protection Agency and Woodrow Wilson International Center for Scholars
Wolfgang Kreyling	GSF National Research Centre for Environment and Health (Germany)
David Lai	U.S. Environmental Protection Agency
Andrew Maynard	Woodrow Wilson International Center for Scholars
Stephen Olin	ILSI Research Foundation/Risk Science Institute
Nancy Monteiro-Riviere	North Carolina State University
David Warheit	DuPont Haskell Laboratory for
	Health and Environmental Sciences
Hong Yang	University of Rochester
